# ZFN-Site searches genomes for zinc finger nuclease target sites and off-target sites

**DOI:** 10.1186/1471-2105-12-152

**Published:** 2011-05-13

**Authors:** Thomas J Cradick, Giovanna Ambrosini, Christian Iseli, Philipp Bucher, Anton P McCaffrey

**Affiliations:** 1 University of Iowa School of Medicine, Department of Internal Medicine, Iowa City, Iowa, 52245, USA; 2 Swiss Institute of Bioinformatics (SIB), Bâtiment Génopode, Université de Lausanne, 1015 Lausanne, Switzerland; 3 Ecole Polytechnique Federale de Lausanne (EPFL), Swiss Institute for Experimental Cancer Research (ISREC), 1015 Lausanne, Switzerland; 4 Ludwig Institute for Cancer Research (LICR), Bâtiment Génopode, Université de Lausanne, 1015 Lausanne, Switzerland

## Abstract

**Background:**

Zinc Finger Nucleases (ZFNs) are man-made restriction enzymes useful for manipulating genomes by cleaving target DNA sequences. ZFNs allow therapeutic gene correction or creation of genetically modified model organisms. ZFN specificity is not absolute; therefore, it is essential to select ZFN target sites without similar genomic off-target sites. It is important to assay for off-target cleavage events at sites similar to the target sequence.

**Results:**

ZFN-Site is a web interface that searches multiple genomes for ZFN off-target sites. Queries can be based on the target sequence or can be expanded using degenerate specificity to account for known ZFN binding preferences. ZFN off-target sites are outputted with links to genome browsers, facilitating off-target cleavage site screening. We verified ZFN-Site using previously published ZFN half-sites and located their target sites and their previously described off-target sites. While we have tailored this tool to ZFNs, ZFN-Site can also be used to find potential off-target sites for other nucleases, such as TALE nucleases.

**Conclusions:**

ZFN-Site facilitates genome searches for possible ZFN cleavage sites based on user-defined stringency limits. ZFN-Site is an improvement over other methods because the FetchGWI search engine uses an indexed search of genome sequences for all ZFN target sites and possible off-target sites matching the half-sites and stringency limits. Therefore, ZFN-Site does not miss potential off-target sites.

## Background

The ability to create double-stranded DNA breaks at specific genomic sequences is important for gene correction therapeutics, targeted gene integration and gene modification for research models as well as gene disruption [1]. Zinc Finger Nucleases (ZFNs) are promising candidates for such specific nucleases. ZFNs consist of the sequence-independent FokI nuclease domain fused to zinc finger proteins (ZFPs). ZFPs can be altered to change their sequence specificity. Cleavage of targeted DNA requires binding of two ZFNs (designated left and right) to adjacent half-sites on opposite strands with correct orientation and spacing, thus forming a FokI dimer [2]. The requirement for dimerization increases ZFN specificity significantly. Three or four finger ZFPs target ~9 or 12 bases per ZFN, or ~18 or 24 bases for the ZFN pair. ZFN pairs have been used for gene targeting at specific genomic loci in insect, plant, animal and human cells [3-10] (and reviewed in [11,12]). Methods are available to measure general ZFN toxicity or the amount of unrepaired DNA ends resulting from ZFN treatment [13-16]; however, determining all possible off-target cleavage sites may be challenging, as some possible cleavage sites can be missed by BLAST and similar methods. ZFN-Site determines the most probable off-target sites for further analysis or testing. Several ZFN design web tools exist that offer BLAST-based searches for potential ZFN off-target sites [17-22]. BLAST searches, which implement a local alignment search, are not optimal for finding ZFN off-target sites and may miss some sites because they utilize seed-based methods with a non-overlapping word index to search only for perfect matches, rather than longer imperfect matches. BLAST also uses an E-value threshold that does not directly correspond to a "# of mismatches" threshold. ZFN-Site is more thorough because it scans one index entry for each nucleotide in the genome, ensuring that no matches are missed. ZFN-Site was created to provide a simple, easy-to-use interface that does not require the end user to possess specialized bioinformatics or search algorithm expertise. ZFN-Site provides an interface that searches multiple genomes for sites with ambiguities, mismatches, multiple spacings, hetero-dimeric binding sites and homo-dimeric binding sites composed of two left or two right ZFN half-sites. Changing these parameters can expand the number of possible off-target sites returned to match the purpose. A larger list enables thorough screening for potential ZFN off-target sites using new methods, such as high-throughput sequencing or mutation screens.

## Implementation

ZFN-Site was developed to quickly locate all possible ZFN target and off-target sites that might be cleaved. Based on the tailoring of search parameters, ZFN-Site generates sets of search strings. To ensure that all sites matching these criteria are found in the requested genomes, ZFN-Site employs the FetchGWI search engine [23]. The input can be either the nucleotide sequence of the intended target site of each ZFN (basic search) or information about each ZFN's binding specificity (relaxed specificity). The number of possible sites is expanded by choice of ZFN spacing, the possibility of ZFN homo-dimerization (see below) and the number of allowed mismatches. The output from ZFN-Site aids in the choice of ZFN pairs that minimize potential off-target sites and allows experimental testing of each ZFN pairs' off-target sites in cells or in mutated animals. Experimentally testing the list of found sites under a series of different conditions may determine the conditions favoring more specific targeting and less off-target cleavage events.

### Basic Target Search

The simplest search method uses the intended target site to scan whole genomes. This type of search is valuable when choosing prospective target sites or when there is no available ZFP mismatch specificity data. ZFN-Site allows searches for off-target sites containing up to two mismatches per half-site. ZFN-Site outputs all target and off-target sites matching the selection criteria.

The genome or genomes to be searched are chosen by clicking on the species list on the left side of the ZFN-Site web page. Scrolling down reveals the full list. Use command-click (mac) or control-click (pc) to choose multiple genomes to be searched simultaneously. A click on ALL searches the entire list of genomes shown in Table 1.

**Table 1:** List of Genomes Scanned by ZFN-Site

Genome Release (Code)	Species
Homo sapiens (HS)	Human
Mus musculus (MM)	Mouse
Danio rerio Zv6 (DR)	Zebrafish
Danio rerio Zv5 (DR5)	Zebrafish
Drosophila melanogaster (DM)	Fruit Fly
Apis mellifera (AME)	Bee
Bos taurus (BT)	Cow
Caenorhabditis elegans NCBIWS170(CE)	Nematode
Canis familiaris (CFA)	Dog
Pan troglodytes (PTR)	Chimpanzee
Rattus norvegicus (RN)	Rat
Saccharomyces cerevisiae (SCE)	Yeast
Tribolium castaneum (TCA)	Beetle
All genomes (ALL)	All of the above

**Figure 1:**
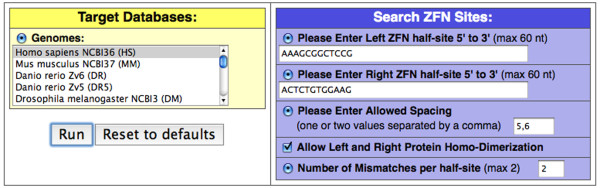
**ZFN-Site genome scan using Basic Target Search**. ZFN-Site search for Sequence 1 using the half-sites described in the text, which are the ZFN target sites found in IL2R-γ [1]. The inputs are set to search the human genome allowing five and six base pair spacing, two mismatches and homo and hetero-dimerization of the half-sites.

Half-sites are entered without spaces, 5' to 3', as they occur on the opposite strand of a ZFN target. The following sequence is an example of the top DNA strand of a three finger ZFN pair target site: 5'-**CGGAGCCGCTTT**aacccACTCTGTGGAAG-3'[3]. The right ZFN half-site is underlined and should be entered into the program 5'-3' as ACTCTGTGGAAG. The left ZFN half-site is the reverse complement of the bold sequence and should be entered 5'-3' as AAAGCGGCTCCG (Figure 1).

The sequence of the DNA spacer between ZFN half-sites (lower case, above) does not greatly influence ZFN specificity, but the length of the spacer between half-sites influences how well a site is cleaved [24]. The allowed number of spacer nucleotides depends on the ZFP-to-FokI linker and is usually five or six nucleotides, although ZFNs with altered linkers have different nucleotide length preferences [25,26]. Genome searches can be run on ZFN-Site with one allowed spacing between half-sites or two spacings if entered separated by a comma (*e.g.*, 5,6). Searches can be repeated using alternate spacings if searching with more than two spacings is required.

In addition to a left ZFN and a right ZFN binding as hetero-dimers, two left or two right ZFNs can bind correctly spaced sites to form homo-dimers and cleave off-target sites [16]. If the "Allow Left and Right Protein Homo-dimerization" box is checked, ZFN-Site also searches for homo-dimeric sites. Use of modified FokI domains may prevent cleavage at most homo-dimeric sites [13,27]. However, identification of homo-dimeric sites and experimental testing for cleavage at each site on these output lists may be necessary to quantitate low levels of cleavage and generate further predictive rules for off-target cleavage events. The specificity of nuclease variants can be experimentally tested using cleavage analysis on the sites comprising the lists of possible off-target sites generated by ZFN-Site [13,25,27-29].

ZFN-Site expands the query targets into a list of queries (or tags) based on the half-sites and inputs. Using increased ambiguities broadens the search. Degenerate nucleotides (specified by standard IUPAC codes) are allowed in the half-site queries because they are then expanded into all possible matching tags. These queries are submitted to an exact search algorithm (described in [23]). The number of such queries increases with the required mismatches and ambiguities (such as Ns and nucleotide IUPAC codes), thus increasing RAM and search time required. Very complex searches may be achieved by breaking the search into parts to speed processing and prevent stalling.

The number of mismatches per half-site (0, 1 or 2) is inputted into the last box. Use 0 to scan only for sites exactly matching the half-sites. This mode is useful for verifying the location of target sites in one or more genomes. The number of off-target sites returned can be greatly increased by allowing 1 or 2 mismatches per half-site. The use of ambiguous nucleotides in the half-sites does not count as a mismatch, and both can be used if needed. Mismatches are allowed in degenerate positions as well. If the user specifies a search with one or two mismatches, ZFN-Site will generate all possible sequence tags that match the target up to the specified number of mismatches.

Once the information above is entered, clicking run will display the query sequences on the next web page, while the genome searches are performed using the FetchGWI program (see paragraph on FetchGWI below). ZFN-Site outputs a list of half-site matches sorted by genome position. This list is scanned by a second program that extracts all combinations on each DNA strand that have the required spacing. For fast performance on the Web, we have limited the number of possible mismatches per ZFN half-site to two. The total number of degenerate nucleotides is also limited to two, such that the computational complexity is manageable.

**Figure 2:**
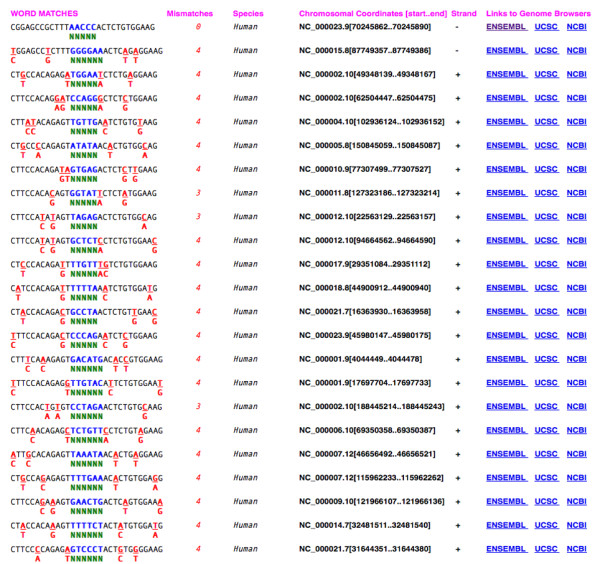
**ZFN-Site Results**. ZFN-Site output listing the IL2R-γ target sequence, in row 1, and other genomic sequences matching the search criteria in Figure 1. Non-matching bases are shown in red below the correct base. Between each pair of target sequences is a spacer with its genomic sequence shown in blue. The number of nucleotides in the spacer is indicated by the number of green Ns. Each sequence row also lists the number of mismatches, chromosomal location, DNA strand and HTML links to their exact location on ENSEMBL, UCSC, NCBI and NCBI browsers. The link to results in text format provides sequences in the list ordered by increasing number of mismatches.

Based on these inputs, ZFN-Site generates a list of genomic sequences that are exact or near-exact matches to the input query set, along with chromosomal coordinates (including NCBI chromosomal accession number and the start and end positions within the chromosome), DNA strand and HTML links to their exact location on ENSEMBL, UCSC and NCBI browsers [23] (Figure 2). Results are output under "WORD MATCHES" in a two-line format for each genomic sequence returned. The top line of each pair of lines depicts the genomic sequence. The lower line displays the differences from the query sequence. Spacer nucleotides are indicated in blue, and in cases where there are ambiguous nucleotides, genomic nucleotides matching an unambiguous portion of the query sequence are in blue. The number of nucleotides in the spacer is indicated by the number of green Ns in the lower line. Red nucleotides depict mismatches. The number of mismatches is displayed, not including positions with degenerate nucleotides (unless mismatches occur at degenerate positions). The next four columns list the matched sequence's "Species", "Chromosomal Coordinates [start..end]", "Strand" and "Links to Genome Browsers". Clicking on the HTML links to the right of a matched genomic sequence will open a browser in either the ENSEMBL, UCSC or NCBI genome browsers. This will direct the user to that exact location, allowing one to identify whether that targeted sequence is in an annotated gene, intron, exon or regulatory sequence.

ZFN-Site can be used to determine if ZFNs may be used to specifically target sites in multiple different genomes. ZFN-Site can scan multiple genomes simultaneously using the same settings or can be run sequentially.

### Relaxed Specificity Search

Previous *in vitro *and cellular ZFP specificity studies may help determine other sequences that may be possibly cleaved by a ZFN pair. This information can come from studies of individual fingers [30-32]. Without Systematic Evolution of Ligands by Exponential Enrichment (SELEX) or similar data (described below), the specificity of a ZFN can be approximated by combining the specificity of the individual fingers, even though this fails to account for the effects of adjacent fingers. There are many manuscripts detailing individual ZFP specificity; non-exhaustive examples include [30-35]. Approximating the specificity of the whole ZFN by compiling the relaxed specificity of the constituent ZFPs may provide more predictive results than using the basic target search, as the individual finger data may help determine the non-specified bases. If there are individual nucleotide positions where the ZFPs can bind several nucleotides, standard IUPAC ambiguity codes should be entered in the half-site.

**Figure 3:**
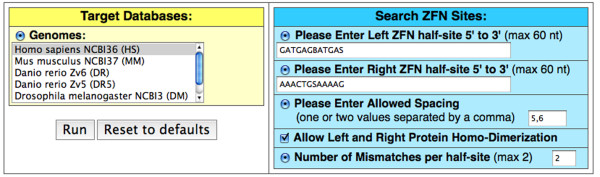
**Benchmarking ZFN-Site against a published CCR5 ZFN off-target analysis**. Previously, Perez *et al*. used SELEX to determine the relaxed specificity of a ZFN pair targeting the CCR5 gene and used this data to scan the genome. We scanned the human genome with ZFN-Site, configured as shown, using the CCR5 ZFN half-sites from Perez *et al*. with ambiguities matching their SELEX data. The bases allowing substitutions are shown in lower case letters. ZFN-Site found each site they listed, paired with their results in Figure 4.

More specific information comes from binding studies of full ZFPs or ZFNs using SELEX. Searches based on experimentally determined specificity are more informative than searches with increased mismatches. If there is SELEX or similar data describing each ZFN's binding specificity, it is also entered in 5' to 3' orientation using standard IUPAC ambiguity codes (as in Figure 3). This allows relaxed specificity searches. For example, a nucleotide in a half-site that can be bound if it is either G or T can be entered as a K. Any non-specified position can be represented by an N (N=A, C, G or T). If scanning with two mismatches, the pair of half-sites should contain less than three ambiguities to prevent computational stalling (see above).

### FetchGWI

ZFN-Site uses FetchGWI to perform rapid and accurate searches of the large sequence databases comprising full genomes. FetchGWI is a C program that relies on pre-computed genome indices and is best used in cases where queries must be mapped very rapidly and efficiently. To get maximal search speed, FetchGWI only searches within the index files that represent the genome sequences. There is one index entry for each nucleotide in the genome. This exhaustive index also ensures that no match can possibly be missed. Other programs, such as BLAST, occasionally scan non-overlapping words and thus can miss possible off-target sites (see below) [20].

### Testing Located Off-Target Sites

Predicted genomic off-target sites should be tested for cleavage. The HTML links are used to download the sequences flanking the site, for use in designing amplification primers for either mutation or sequence analysis. The listed potential off-target sites can be assayed by PCR and mutation detection [7] or deep sequencing [5] to determine ZFN specificity.

If ZFN-Site locates more sites that match the selected criteria than can be tested, the criteria may be narrowed by using less mismatches or using less ambiguous nucleotides for relaxed searches. The list of found sites can also be narrowed using the text output. If the text output link is clicked, the found sites are outputted in another screen in order of increasing number of total mismatches. If a search is conducted using two mismatches per half-site, the output can be greatly narrowed by selecting the genomic sequences at the top of the list with three or fewer total mismatches.

This list of possible target sequences can be further analysed using other computer programs. For example, the output can be ranked using an excel spreadsheet containing a positional weight matrix based on experimentally determined specificity data as described below.

## Results

**Figure 4:**
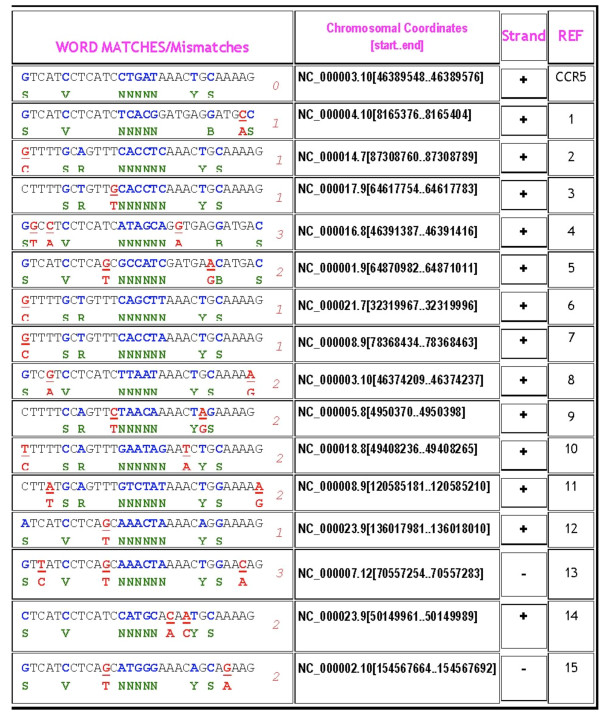
**ZFN-Site returns sites found in previous CCR5 ZFN off-target analysis**. The sequences returned by ZFN-Site were matched to the sequences found by Perez *et al*. For clarity of presentation, the ZFN-Site output was arranged to match the order of Perez *et al*. ZFN-Site found all the sites found by the unpublished algorithm of Perez *et al.*, thus validating ZFN-Site. We replaced the column containing the genome browser links with a column referencing the order listed in the first column of Perez, *et al*. Columns detail the human sequences matching the query, the chromosomal coordinates and the strand. The first row consists of the exact match to the CCR5 genomic sequence.

ZFN-Site was validated by comparing our results to a previously published study by Perez *et al. *[7]. Perez *et al. *looked for off-target cleavage by a pair of ZFNs specific for the gene coding for human C-C chemokine receptor type 5 (CCR5). This study used an unpublished algorithm to identify potential off-target sites by scanning the human genome using *in vitro *SELEX selection specificity data [7]. Their sequencing of the identified off-target sites revealed that a site in the related CCR2 gene was also cleaved at a low frequency. The left and right ZFN half-sites, including ambiguities suggested from their SELEX data, were compiled and entered into ZFN-Site (Figure 3). ZFN-Site found the CCR5 target site and each of the off-target sites on their list, including the experimentally verified CCR2 off-target cleavage site (Figure 4). Additional file 1, Figure S1 contains ZFN-Site output with less than three total mismatches.

**Figure 5:**
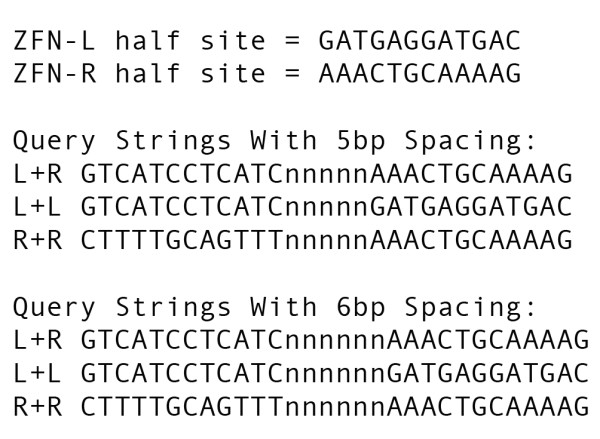
**ZFN half-sites and resulting query sequences with 5 or 6 bp spacing**. ZFN-Site generates six query sequences based on the two half-sites entered for homo-dimerization, and two different spacings are allowed between the half-sites. The left and right half-sites are listed followed by the resulting query sequences if the half-sites are separated by 5 or 6 bps. Each list includes a query string made of one left and one right half-site (L + R), two left half-sites (L + L) and two right half-sites (R + R). Each of these would need to be searched individually if using BLAST.

Multiple BLAST searches sometimes accomplish the same function as ZFN-Site if one inputs all possible permutations of homo/heterodimers, spacings and relaxed specificities. This can be labor-intensive. For example, six BLAST queries for permutations of the Perez *et al*. ZFNs could replace one ZFN-Site search without ambiguities (Figure 5). However, in contrast to ZFN-Site, BLAST does not allow ambiguous bases. While BLAST could return these sites, user intervention would be required to distinguish these from true mismatches. ZFN-Site thus simplifies the process of searching for ZFN off-target sites.

ZFN-Site locates every site matching the specified search criteria. In contrast, it has been noted that the BLAST methodology may not find every ZFN site [20]. Because BLAST searches implement a local alignment search, they are incapable of reproducing the same type of results as ZNF-Site. To compare results to the single ZFN-Site search above, six sets of BLAST searches for the CCR5 ZFN pair were done to include homo- and hetero-dimerization at both 5 bp and 6 bp spacing. Some of the sites found by Perez *et al*. and by ZFN-Site were not found using BLAST, although the BLAST parameters were optimized to attempt to return all matches (Additional file 2, Figure S2). The BLAST search for the right homo-dimer pair with six base spacing failed to return two sequences found by Perez *et al*. and ZFN-Site (numbers 10 and 11). This search returned 474 genomic sequences, many of which were too dissimilar to be likely off-target sites. Because BLAST outputs the matching portion of the sequences with the ends truncated, further user intervention was required to verify the total similarity of these sequences.

In some cases, ZFN-Site may return a large number of sequences. The degree to which one may wish to narrow a list of ZFN-Site outputs depends on the experimental means used to search for off-target cleavage and the resources for scanning multiple sites. The use of deep sequencing may require less narrowing of the list because one can quantitatively test hundreds of sites. Until more information is available on the actual prevalence of ZFN off- target cleavage, it would be desirable to test as many potential off-target sites as experimentally feasible.

A post-processing step using positional weight matrices (PWM) can be used to rank the output of ZFN-Site. Additional file 3 is an example of a spreadsheet used to rank ZFN-Site output using PWMs based on the graph of nucleotide frequencies in Perez *et al*. [7]. The top putative target sites could then be tested experimentally.

## Conclusions

ZFN-Site is applicable to genome searches for pairs of half-sites in nucleases or other types of DNA binding proteins. Here, we have presented a user friendly interface allowing a directed search of multiple genomes and have validated its use for finding ZFN sites and off-target sites in the human genome. Experimental testing for ZFN cleavage at the potential sites found by ZFN-Site using large scale sequencing or mutation detection may provide a more thorough understanding of the determinants of ZFN specificity and allow optimization for decreased off-target cleavage events. These results can also be compared with results from other methods for detecting off-target cleavage and toxicity [13-16].

Recently, other nucleases, such as TALE nucleases, have been used for genome alteration [36-39]. While ZFN-Site was tailored to locate ZFN off-target sites, it can also be used to find targets for TALE nucleases. A spreadsheet for creating PWMs and ranking output for TALE nucleases is available upon request.

## Authors' contributions

TJC provided the initial concept, methods and pseudo-code. GA redesigned the querying methods and implemented the Web interface. TJC tested and benchmarked early versions and provided spreadsheet and supplemental files. CI developed the FetchGWI interface with contribution from GA. TJC & APM wrote the manuscript with contributions from GA and PB.

## Availability and requirements

ZFN-Site is available freely on our web site, http://ccg.vital-it.ch/tagger/targetsearch.html[40], and the FetchGWI source code is also available at Source Forge, http://sourceforge.net/projects/tagger/[41].

Project name: ZFN-Site

Project home page: http://ccg.vital-it.ch/tagger/targetsearch.html

Operating system(s): Platform independent

Programming language: C and Perl

Other requirements: None

License: GNU General Public License (GPL), version 2

Any restrictions to use by non-academics: No

## Supplementary Material

Additional File 1 **Figure S1 - Genomic sites located by ZFN-Site with up to three mismatches**. ZFN-Site was run using two mismatches and two ambiguities per half-site as in Figure 3. Genomic sites were located that matched each site found by Perez *et al*. [7] as shown in Figure 4. This comparison provides validation for ZFN-Site. Numerous other sites not described in Perez *et al*. were also found by ZFN-Site, and these can be analyzed experimentally in order to determine if they are actual off-target sites. The text output was sorted by increasing number of mismatches for each genomic location. This is the full list of genomic sequence with three or fewer total mismatches from the half-sites. Mis, # of mismatches; Ch, chromosome; strand, DNA strand Click here for file

Additional File 2 **Figure S2 - BLAST search failed to return the full list of potential off-target sites**. Because BLAST searches implement a local alignment search, they are incapable of reproducing the same type of results as ZNF-Site; this is demonstrated by the output of one BLAST search that failed to find some off-target sites but returned many irrelevant sites. BLAST searches were run using each of the six half-site combinations from Perez *et al*. [7]. This figure shows the results from the BLAST search consisting of two right half-sites separated by six bases (CTTTTGCAGTTT nnnnnn AAACTGCAAAAG). To increase the likelihood of returning all relevant sequences, the EXPECT parameter was raised to a low stringency value of 100, and the penalty for a nucleotide mismatch was dropped to -1. Of the six sequences of this type previously located by Perez *et al*. and by ZFN-Site (Figure 4), BLAST did not locate two sequences (sequences 10 and 11 from Perez *et al*. [7]). BLAST did locate four of the six sequences (sequences 2, 3, 6 and 7) and six similar sequences but also returned 474 sequences that were dissimilar enough to be unlikely to mediate ZFN cleavage. BLAST returns matches in both the forward and reverse DNA strand as indicated in the far right column. The fifth column contains a comparison of the BLAST result to the reference sequence. Mismatches are indicated by an A, C, G or T. A mismatched base not returned by BLAST is shown by an X. Bases truncated at the end of the query sequence are show by a "?", as the user would have to refer back to the genomic sequence to determine if the bases indicated by "?" matched the query sequence, unlike in ZFN-Site. Because BLAST uses a strictly local alignment algorithm, non-matching ends are automatically truncated from the query in order to keep the total number of mismatches low. With the mismatch penalty used in this search, the percent difference threshold for truncating ends is 50%. This figure shows that potential off-target sites can be found using BLAST, but BLAST misses some potential off-target sites. BLAST returns many extraneous sites requiring evaluation through additional processing steps and is much more cumbersome to use than ZFN-Site. Chrom, chromosomal location Click here for file

Additional File 3 **Example of a spreadsheet for ranking ZFN-Site output**. The first tab in the spreadsheet provides instruction for ranking ZFN-Site text output based on specificity data. SELEX data are converted to positional weight matrices (PWM). Where base frequencies were zero, a very small, arbitrary pseudocount factor was used to prevent multiplication by zero. PWM were used to score the half-sites of each genomic sequence, assign the matching target sequence and compute the ranking score. The genomic sequences were ranked based on these numbers. Sorting by these scores allowed the choice of sequences most similar to the specificity data. Click here for file
