# Supplementary material for: ZFN-Site searches genomes for zinc finger nuclease target sites and off-target sites
Source: BMC Bioinformatics. 2011 May 13;12:152. doi: 10.1186/1471-2105-12-152 (PMC3113941; doi:10.1186/1471-2105-12-152)
Supplement: Additional File 1 — Figure S1 - Genomic sites located by ZFN-Site with up to three mismatches. ZFN-Site was run using two mismatches and two ambiguities per half-site as in Figure 3. Genomic sites were located that matched each site found by Perez et al. [7] as shown in Figure 4. This comparison provides validation for ZFN-Site. Numerous other sites not described in Perez et al. were also found by ZFN-Site, and these can be analyzed experimentally in order to determine if they are actual off-target sites. The text output was sorted by increasing number of mismatches for each genomic location. This is the full list of genomic sequence with three or fewer total mismatches from the half-sites. Mis, # of mismatches; Ch, chromosome; strand, DNA strand [file 1471-2105-12-152-S1.PDF]

Word Matches	Query	Mis	Ch	Genome Coordinates	Str	Genome
GTCATCCTCATCCTGATAAACTGCAAAAG	STCATVCTCATCNNNNNAAACTGSAAAAG	0	3	NC_000003.10[46389548..46389576]	+	Human
GTCATCCTCATCTCACGGATGAGGATGCC	STCATVCTCATCNNNNNGATGAGBATGAS	1	4	NC_000004.10[8165376..8165404]	+	Human
GTTTTGCAGTTTCACCTCAAACTGCAAAAG	CTTTTSCAGTTTNNNNNNAAACTGSAAAAG	1	14	NC_000014.7[87308760..87308789]	+	Human
GTCGTCCTCATCTTAATAAACTGCAAAAA	STCATVCTCATCNNNNNAAACTGSAAAAG	2	3	NC_000003.10[46374209..46374237]	+	Human
CTCATCCTCATCCATGCACAATGCAAAAG	STCATVCTCATCNNNNNAAACTGSAAAAG	2	X	NC_000023.9[50149961..50149989]	+	Human
CTCATGCTCATCATTTGTTAACTGCAAAAT	STCATVCTCATCNNNNNNAAACTGSAAAAG	2	21	NC_000021.7[34098210..34098239]	-	Human
GTCATCCTCAGCGCCATCGATGAACATGAC	STCATVCTCATCNNNNNNGATGAGBATGAS	2	1	NC_000001.9[64870982..64871011]	+	Human
GTCATCCTCATCTTCATCCATGAGGAAGAG	STCATVCTCATCNNNNNNGATGAGBATGAS	2	3	NC_000003.10[185963690..185963719	+	Human
CTCTTGCTCATCCTGTTGGATCAGCATGAC	STCATVCTCATCNNNNNNGATGAGBATGAS	2	5	NC_000005.8[142090919..142090948]	+	Human
CTTTTCCAGTTCTAACAAAACTAGAAAAG	CTTTTSCAGTTTNNNNNAAACTGSAAAAG	2	5	NC_000005.8[4950370..4950398]	+	Human
GTTTTGCTGTTTCACCTAAAACTGCAAAAG	CTTTTSCAGTTTNNNNNNAAACTGSAAAAG	2	8	NC_000008.9[78368434..78368463]	+	Human
CTTATGCAGTTTGTCTATAAACTGGAAAAA	CTTTTSCAGTTTNNNNNNAAACTGSAAAAG	2	8	NC_000008.9[120585181..120585210]	+	Human
CTTTTGCTGTTGCACCTCAAACTGCAAAAG	CTTTTSCAGTTTNNNNNNAAACTGSAAAAG	2	17	NC_000017.9[64617754..64617783]	+	Human
TTTTTCCAGTTTGAATAGAATCTGCAAAAG	CTTTTSCAGTTTNNNNNNAAACTGSAAAAG	2	18	NC_000018.8[49408236..49408265]	+	Human
GTTTTGCTGTTTCAGCTTAAACTGCAAAAG	CTTTTSCAGTTTNNNNNNAAACTGSAAAAG	2	21	NC_000021.7[32319967..32319996]	+	Human
CTCATACTCATTTTACCAAACAGGAAAAT	STCATVCTCATCNNNNNAAACTGSAAAAG	3	1	NC_000001.9[170736257..170736285]	+	Human
GTCCTGCTCAGCAAAAGAAACTGAAAAAG	STCATVCTCATCNNNNNAAACTGSAAAAG	3	6	NC_000006.10[52114315..52114343]	+	Human
GTTTTCCTCATCAAAGCAAACTGCAAAAT	STCATVCTCATCNNNNNAAACTGSAAAAG	3	11	NC_000011.8[13441738..13441766]	+	Human
CTCCTCCTCTTCCTTGAAAACTGCAGAAG	STCATVCTCATCNNNNNAAACTGSAAAAG	3	11	NC_000011.8[68689498..68689526]	+	Human
CTGAGCCTCATCATCCTAAACTGCAAAAC	STCATVCTCATCNNNNNAAACTGSAAAAG	3	11	NC_000011.8[121312904..121312932]	+	Human
CTCATGATCATCCATTGTAACTGGAAGAG	STCATVCTCATCNNNNNAAACTGSAAAAG	3	12	NC_000012.10[487470..487498]	+	Human
CTCATGCTCATTTAAAAAAACTGAAATAG	STCATVCTCATCNNNNNAAACTGSAAAAG	3	13	NC_000013.9[71375317..71375345]	+	Human
TTCATAATCATCTAAATAAACTGGAAAAA	STCATVCTCATCNNNNNAAACTGSAAAAG	3	15	NC_000015.8[19977449..19977477]	+	Human
CTCATATTCATCCTTCCAAACAGGAAAAC	STCATVCTCATCNNNNNAAACTGSAAAAG	3	15	NC_000015.8[25854714..25854742]	+	Human
GTCATACTCATGGTCTCAAACTGCAGAGG	STCATVCTCATCNNNNNAAACTGSAAAAG	3	15	NC_000015.8[83928170..83928198]	+	Human
CTCATCTTCAACACAGAAAACTAGAAAAG	STCATVCTCATCNNNNNAAACTGSAAAAG	3	16	NC_000016.8[46459781..46459809]	+	Human
CTAATCCTCATCTGTAAATACTGAAAAAG	STCATVCTCATCNNNNNAAACTGSAAAAG	3	1	NC_000001.9[183621707..183621735]	-	Human
GTGATCCTCATTCCAGTAAACTGGAAAAC	STCATVCTCATCNNNNNAAACTGSAAAAG	3	1	NC_000001.9[238548832..238548860]	-	Human
GTCATCCTCAGCATGGGAAACAGCAGAAG	STCATVCTCATCNNNNNAAACTGSAAAAG	3	2	NC_000002.10[154567664..154567692	-	Human
CTCATAGTCAACATAGGAAATTGGAAAAG	STCATVCTCATCNNNNNAAACTGSAAAAG	3	3	NC_000003.10[128288918..128288946	-	Human
CTCATCCTCACCAGGGAAAAGAGGAAAAG	STCATVCTCATCNNNNNAAACTGSAAAAG	3	3	NC_000003.10[153634491..153634519	-	Human
GTCACACTCATCTAAACAATCTGGAAAAA	STCATVCTCATCNNNNNAAACTGSAAAAG	3	6	NC_000006.10[102530888..102530916	-	Human
CTCCTCCACATCCTTTTAAACTAGAAAAG	STCATVCTCATCNNNNNAAACTGSAAAAG	3	8	NC_000008.9[17059268..17059296]	-	Human
CACATACTCATTGATCTAAACTGGGAAAG	STCATVCTCATCNNNNNAAACTGSAAAAG	3	8	NC_000008.9[88998566..88998594]	-	Human
GGCCTCCTCATCTCTTTAAACTGGAAATG	STCATVCTCATCNNNNNAAACTGSAAAAG	3	12	NC_000012.10[74249717..74249745]	-	Human
GTCATCTTCATCAAAAGGAACTGCAAAAC	STCATVCTCATCNNNNNAAACTGSAAAAG	3	17	NC_000017.9[61624429..61624457]	-	Human
CCCAAACTCATCCTTAAGAACTGCAAAAG	STCATVCTCATCNNNNNAAACTGSAAAAG	3	21	NC_000021.7[43918421..43918449]	-	Human
CCCATCCTCATCCCTGGAACCTGCAAATG	STCATVCTCATCNNNNNAAACTGSAAAAG	3	X	NC_000023.9[117533563..117533591]	-	Human
GTCATCCTCAGCAAACTAACACAGGAAAAG	STCATVCTCATCNNNNNNAAACTGSAAAAG	3	2	NC_000002.10[63482829..63482858]	+	Human
CTCTTACTCATTAAATACATACTGCAAAAG	STCATVCTCATCNNNNNNAAACTGSAAAAG	3	3	NC_000003.10[84155880..84155909]	+	Human
TTCATGCTCATCACTTCAAAACTGTAGAAG	STCATVCTCATCNNNNNNAAACTGSAAAAG	3	5	NC_000005.8[14228053..14228082]	+	Human
CTCATGCACATCATAGTAAAACTGACAAAG	STCATVCTCATCNNNNNNAAACTGSAAAAG	3	7	NC_000007.12[85045662..85045691]	+	Human
CTCCTGCTCCTCGGGAACAAACTGGAACAG	STCATVCTCATCNNNNNNAAACTGSAAAAG	3	11	NC_000011.8[72163422..72163451]	+	Human
GTCATCCCAATCGAAGAAAAACTGAAAAAG	STCATVCTCATCNNNNNNAAACTGSAAAAG	3	12	NC_000012.10[33484433..33484462]	+	Human
GTCATCCGCATCGCCCTGGAACTGGAAAAA	STCATVCTCATCNNNNNNAAACTGSAAAAG	3	14	NC_000014.7[64329872..64329901]	+	Human
CTCATACTCCTCCAATAGAAAATTGAAAAG	STCATVCTCATCNNNNNNAAACTGSAAAAG	3	15	NC_000015.8[84299921..84299950]	+	Human
GTCATCCTCATTATTCATAAAATGGAAAAC	STCATVCTCATCNNNNNNAAACTGSAAAAG	3	X	NC_000023.9[67948464..67948493]	+	Human
ATCATCCTCAGCAAACTAAAACAGGAAAAG	STCATVCTCATCNNNNNNAAACTGSAAAAG	3	X	NC_000023.9[136017981..136018010]	+	Human

GTGATACTCATCATCAGCAATCTGCATAAG	STCATVCTCATCNNNNNNAAACTGSAAAAG	3	1	NC_000001.9[99456616..99456645]	-	Human
CTGATACTAATCAACTCAAGACTGCAAAAG	STCATVCTCATCNNNNNNAAACTGSAAAAG	3	3	NC_000003.10[146224167..146224196	-	Human
CTCATCCCCATCACATTAAAACTGAAAAAT	STCATVCTCATCNNNNNNAAACTGSAAAAG	3	5	NC_000005.8[74448316..74448345]	-	Human
GTTATCCTCAGCAAACTAAAACTGGAACAG	STCATVCTCATCNNNNNNAAACTGSAAAAG	3	7	NC_000007.12[70557254..70557283]	-	Human
GTCAACCTCAACACCTACAGACTGCAAAAG	STCATVCTCATCNNNNNNAAACTGSAAAAG	3	9	NC_000009.10[80584200..80584229]	-	Human
CTCATGCTCAAGTAATTTAAACTTGAAAAG	STCATVCTCATCNNNNNNAAACTGSAAAAG	3	9	NC_000009.10[82540310..82540339]	-	Human
CACATACTCAGCCTTCGAAAACTTCAAAAG	STCATVCTCATCNNNNNNAAACTGSAAAAG	3	10	NC_000010.9[2465786..2465815]	-	Human
ATCATCCTCAACAAACTAAAACAGGAAAAG	STCATVCTCATCNNNNNNAAACTGSAAAAG	3	10	NC_000010.9[54268729..54268758]	-	Human
CACATGTTCATCGCTTTTAAAATGCAAAAG	STCATVCTCATCNNNNNNAAACTGSAAAAG	3	10	NC_000010.9[119506054..119506083]	-	Human
GTCATCCTCAGCAAATTAACACAGGAAAAG	STCATVCTCATCNNNNNNAAACTGSAAAAG	3	12	NC_000012.10[14242609..14242638]	-	Human
GTCATACGCATACAAATCAAACTGCAAAAA	STCATVCTCATCNNNNNNAAACTGSAAAAG	3	15	NC_000015.8[91242250..91242279]	-	Human
CTCAGGCTCAGCTTACTGAAACTGGAAAAC	STCATVCTCATCNNNNNNAAACTGSAAAAG	3	16	NC_000016.8[61880221..61880250]	-	Human
CTCATATACATCACTAATAAACTGGAATAG	STCATVCTCATCNNNNNNAAACTGSAAAAG	3	18	NC_000018.8[32678726..32678755]	-	Human
ATTATACTCATCTTACTAATGAGTATGAG	STCATVCTCATCNNNNNGATGAGBATGAS	3	1	NC_000001.9[26789962..26789990]	+	Human
GTCATAATCATGATGATGATGAGGATGAT	STCATVCTCATCNNNNNGATGAGBATGAS	3	2	NC_000002.10[24534455..24534483]	+	Human
CTCATATTCTTCAAAGAGCTGAGTATGAC	STCATVCTCATCNNNNNGATGAGBATGAS	3	2	NC_000002.10[145077608..145077636	+	Human
GTCATCCTCAACGTAATGTTGAGTTTGAC	STCATVCTCATCNNNNNGATGAGBATGAS	3	2	NC_000002.10[228923897..228923925	+	Human
GTTATCTTCATCTTCATGTTGAGGATGAG	STCATVCTCATCNNNNNGATGAGBATGAS	3	3	NC_000003.10[191031982..191032010	+	Human
ATCATCCTCATCTTGCAGATGAGGGAGAC	STCATVCTCATCNNNNNGATGAGBATGAS	3	5	NC_000005.8[154350437..154350465]	+	Human
CTCACCCTCATCGGGATTATGGGTATGAC	STCATVCTCATCNNNNNGATGAGBATGAS	3	7	NC_000007.12[38643008..38643036]	+	Human
CTCATCCCCATCTTGAAGATGAGGAAAAC	STCATVCTCATCNNNNNGATGAGBATGAS	3	8	NC_000008.9[75690181..75690209]	+	Human
ATCTTCCTCATCTGTAAGATGGGGATGAC	STCATVCTCATCNNNNNGATGAGBATGAS	3	10	NC_000010.9[46423750..46423778]	+	Human
GTTATCCTCATTTTATAGATGAGGCTGAG	STCATVCTCATCNNNNNGATGAGBATGAS	3	11	NC_000011.8[78575155..78575183]	+	Human
GTTATATTCATCAAAGAGATGAATATGAG	STCATVCTCATCNNNNNGATGAGBATGAS	3	12	NC_000012.10[104203242..104203270	+	Human
CTCATCCCCATCATACACATGAGGAAGAC	STCATVCTCATCNNNNNGATGAGBATGAS	3	13	NC_000013.9[109749912..109749940]	+	Human
GTTATCCTCATCTACCGCATGAGCATGTG	STCATVCTCATCNNNNNGATGAGBATGAS	3	16	NC_000016.8[55222132..55222160]	+	Human
CTGATCCTCATCTTTAAAATGAGGATAAG	STCATVCTCATCNNNNNGATGAGBATGAS	3	18	NC_000018.8[34171315..34171343]	+	Human
CTCATATTCATCATATAGATGAAGAAGAG	STCATVCTCATCNNNNNGATGAGBATGAS	3	18	NC_000018.8[50055984..50056012]	+	Human
CTTATCCTCATTTTAGAGACGAGCATGAG	STCATVCTCATCNNNNNGATGAGBATGAS	3	20	NC_000020.9[40929285..40929313]	+	Human
CTAAAACTCATCTTTCCAATGAGCATGAG	STCATVCTCATCNNNNNGATGAGBATGAS	3	20	NC_000020.9[53521709..53521737]	+	Human
GTCATCCCCATCTTGCAGATGGGGCTGAG	STCATVCTCATCNNNNNGATGAGBATGAS	3	X	NC_000023.9[20445897..20445925]	+	Human
TTCATCCTCATGATTCTGGTGAGGATGAC	STCATVCTCATCNNNNNGATGAGBATGAS	3	X	NC_000023.9[39130101..39130129]	+	Human
CTCATATTCATCAGTTAGATGTGCATTAC	STCATVCTCATCNNNNNGATGAGBATGAS	3	Y	NC_000024.8[13881043..13881071]	+	Human
GTCATGCTGATTGAACAAGATGAGCAAGAG	STCATVCTCATCNNNNNNGATGAGBATGAS	3	1	NC_000001.9[86240905..86240934]	+	Human
CACATGCTCATCCCCCAGGCTGAGTATGGG	STCATVCTCATCNNNNNNGATGAGBATGAS	3	1	NC_000001.9[166474850..166474879]	+	Human
CTCATACTCTTCTTCACTGATGAGCAGGAA	STCATVCTCATCNNNNNNGATGAGBATGAS	3	3	NC_000003.10[122634125..122634154	+	Human
CTCACCCTCATCCCATATGATGAAGATAAC	STCATVCTCATCNNNNNNGATGAGBATGAS	3	4	NC_000004.10[138126738..138126767	+	Human
GTCACACGCATCAACGCCGATGAGGCTGAG	STCATVCTCATCNNNNNNGATGAGBATGAS	3	5	NC_000005.8[173067623..173067652]	+	Human
GTCATCCTCATTGGCAAAGATGATGATGCC	STCATVCTCATCNNNNNNGATGAGBATGAS	3	5	NC_000005.8[178351036..178351065]	+	Human
CACATCCTCATTCTGCCTGATGAGCCTGAC	STCATVCTCATCNNNNNNGATGAGBATGAS	3	6	NC_000006.10[126171027..126171056	+	Human
CTTATCCTCCTCCCCTTAGATAAGGATGAC	STCATVCTCATCNNNNNNGATGAGBATGAS	3	15	NC_000015.8[23067639..23067668]	+	Human
GGCCTCCTCATCATAGCAGGTGAGGATGAC	STCATVCTCATCNNNNNNGATGAGBATGAS	3	16	NC_000016.8[46391387..46391416]	+	Human
CACATCCTCATCAGCTTCGATGATCATCAG	STCATVCTCATCNNNNNNGATGAGBATGAS	3	20	NC_000020.9[34092604..34092633]	+	Human
GTCTTCATCATCAGGTTGGGTGAGGATGAG	STCATVCTCATCNNNNNNGATGAGBATGAS	3	22	NC_000022.9[32002764..32002793]	+	Human
CTCCTCCTCAGCCTCCTCAATGAGGATGAG	STCATVCTCATCNNNNNNGATGAGBATGAS	3	X	NC_000023.9[8002875..8002904]	+	Human
CGCTTGCTCATCCCTGGGGATGTGGATGAG	STCATVCTCATCNNNNNNGATGAGBATGAS	3	X	NC_000023.9[25272687..25272716]	+	Human
TTCACGCTCATCCTGCAGAATGAGCATGAC	STCATVCTCATCNNNNNNGATGAGBATGAS	3	X	NC_000023.9[148093895..148093924]	+	Human
CTTTTGGAGTTTTATTGGAACTGGAGAAG	CTTTTSCAGTTTNNNNNAAACTGSAAAAG	3	1	NC_000001.9[184895032..184895060]	+	Human
CTTTTCCTGTTTTATAAAAACTCCAAAAA	CTTTTSCAGTTTNNNNNAAACTGSAAAAG	3	2	NC_000002.10[64665861..64665889]	+	Human

TTTTTCCAGTTTCCATAAAACTGGAAGAA	CTTTTSCAGTTTNNNNNAAACTGSAAAAG	3	3	NC_000003.10[77325644..77325672]	+	Human
CTTTTCTAGTTCTAACAAAACTAGAAAAG	CTTTTSCAGTTTNNNNNAAACTGSAAAAG	3	5	NC_000005.8[4950282..4950310]	+	Human
CTTTTGCTGTTTAGCGAAAATAGCAAAAG	CTTTTSCAGTTTNNNNNAAACTGSAAAAG	3	5	NC_000005.8[172124493..172124521]	+	Human
CTTTTGGAGTTTTCAAGAAAATGCAACAG	CTTTTSCAGTTTNNNNNAAACTGSAAAAG	3	6	NC_000006.10[92162074..92162102]	+	Human
AGTTTGCAGTTTCTTCGAAAATGCAAAAG	CTTTTSCAGTTTNNNNNAAACTGSAAAAG	3	7	NC_000007.12[142309426..142309454	+	Human
GTTTTACAGTTTTGTTAAAACTTGAAAAG	CTTTTSCAGTTTNNNNNAAACTGSAAAAG	3	7	NC_000007.12[158177155..158177183	+	Human
CTTTTTCAGTTTTTAAAAAACAGCAAAAT	CTTTTSCAGTTTNNNNNAAACTGSAAAAG	3	9	NC_000009.10[97709133..97709161]	+	Human
CATTTGCAGTTTGCATCAAATTGGAAAAT	CTTTTSCAGTTTNNNNNAAACTGSAAAAG	3	10	NC_000010.9[131322626..131322654]	+	Human
CTTTTGCATTTCAAAATAAACAGGAAAAG	CTTTTSCAGTTTNNNNNAAACTGSAAAAG	3	11	NC_000011.8[108275333..108275361]	+	Human
GTTTTGCAGTTTGAAGTGCAACAGCAAAAG	CTTTTSCAGTTTNNNNNNAAACTGSAAAAG	3	1	NC_000001.9[80506099..80506128]	+	Human
CTTTTGTAGTTTGAGGTGCAACAGCAAAAG	CTTTTSCAGTTTNNNNNNAAACTGSAAAAG	3	1	NC_000001.9[168130804..168130833]	+	Human
ATTTTGCAGTTTGAGGTGCAACAGCAAAAG	CTTTTSCAGTTTNNNNNNAAACTGSAAAAG	3	2	NC_000002.10[35746080..35746109]	+	Human
TTTTTGCAGTCTCTCAGTAAACTGCCAAAG	CTTTTSCAGTTTNNNNNNAAACTGSAAAAG	3	4	NC_000004.10[129495361..129495390	+	Human
CTTTTCCATTTTTGCAAAAAAATGCAAAAT	CTTTTSCAGTTTNNNNNNAAACTGSAAAAG	3	5	NC_000005.8[14989535..14989564]	+	Human
GTTTTCCAGTTTCTTGAAAAACTAGAAAAA	CTTTTSCAGTTTNNNNNNAAACTGSAAAAG	3	5	NC_000005.8[153542846..153542875]	+	Human
GTTTTGCAGTTGTGCCTCAATCTGCAAAAG	CTTTTSCAGTTTNNNNNNAAACTGSAAAAG	3	7	NC_000007.12[51237498..51237527]	+	Human
CTTTTCCAATTTATGTAGAAACTGCAAGAA	CTTTTSCAGTTTNNNNNNAAACTGSAAAAG	3	7	NC_000007.12[76694346..76694375]	+	Human
CTTTTGTAGTTTGAAATGAGACAGCAAAAG	CTTTTSCAGTTTNNNNNNAAACTGSAAAAG	3	8	NC_000008.9[40764088..40764117]	+	Human
CTTTTTCAGTTTAAAATTAAGCTGGAAAAT	CTTTTSCAGTTTNNNNNNAAACTGSAAAAG	3	8	NC_000008.9[63860905..63860934]	+	Human
CTATTCCAGTTTATAATAAAGCTGGAAAAA	CTTTTSCAGTTTNNNNNNAAACTGSAAAAG	3	14	NC_000014.7[29959176..29959205]	+	Human
ATTTTGCAGTTTGGCAGCAAACTGCAAAGA	CTTTTSCAGTTTNNNNNNAAACTGSAAAAG	3	15	NC_000015.8[23419228..23419257]	+	Human
CTTTTGCAGTTAGAGGTGCAACTGCAAAAC	CTTTTSCAGTTTNNNNNNAAACTGSAAAAG	3	X	NC_000023.9[29275290..29275319]	+	Human
CTTCTGCAATTTCTAGGCAAACTGGAAAAC	CTTTTSCAGTTTNNNNNNAAACTGSAAAAG	3	X	NC_000023.9[107295889..107295918]	+	Human
CTTTTCCAGTAAATTTGAAAACTGGAAAAA	CTTTTSCAGTTTNNNNNNAAACTGSAAAAG	3	X	NC_000023.9[132286298..132286327]	+	Human
