# Supplementary material for: ZFN-Site searches genomes for zinc finger nuclease target sites and off-target sites
Source: BMC Bioinformatics. 2011 May 13;12:152. doi: 10.1186/1471-2105-12-152 (PMC3113941; doi:10.1186/1471-2105-12-152)
Supplement: Additional File 2 — Figure S2 - BLAST search failed to return the full list of potential off-target sites. Because BLAST searches implement a local alignment search, they are incapable of reproducing the same type of results as ZNF-Site; this is demonstrated by the output of one BLAST search that failed to find some off-target sites but returned many irrelevant sites. BLAST searches were run using each of the six half-site combinations from Perez et al. [7]. This figure shows the results from the BLAST search consisting of two right half-sites separated by six bases (CTTTTGCAGTTT nnnnnn AAACTGCAAAAG). To increase the likelihood of returning all relevant sequences, the EXPECT parameter was raised to a low stringency value of 100, and the penalty for a nucleotide mismatch was dropped to -1. Of the six sequences of this type previously located by Perez et al. and by ZFN-Site (Figure 4), BLAST did not locate two sequences (sequences 10 and 11 from Perez et al. [7]). BLAST did locate four of the six sequences (sequences 2, 3, 6 and 7) and six similar sequences but also returned 474 sequences that were dissimilar enough to be unlikely to mediate ZFN cleavage. BLAST returns matches in both the forward and reverse DNA strand as indicated in the far right column. The fifth column contains a comparison of the BLAST result to the reference sequence. Mismatches are indicated by an A, C, G or T. A mismatched base not returned by BLAST is shown by an X. Bases truncated at the end of the query sequence are show by a "?", as the user would have to refer back to the genomic sequence to determine if the bases indicated by "?" matched the query sequence, unlike in ZFN-Site. Because BLAST uses a strictly local alignment algorithm, non-matching ends are automatically truncated from the query in order to keep the total number of mismatches low. With the mismatch penalty used in this search, the percent difference threshold for truncating ends is 50%. This figure shows that potential off-target sites can be [file 1471-2105-12-152-S2.PDF]

Chrom	Subject	E value	Mismatches	Seq. #
NC_000014.7	TTTTGCAGTTTCACCTCAAACTGCAAAAG	38	X-----------nnnnnn------------	2 For.
NC_000014.7	CTTTTGCAGTTTGAGGTGAAACTGCAAAA	38	------------nnnnnn----------X	2 Rev.
NC_000014.7	CTTTTGCAGTTTGAGGCAAGACAGCAAAA	31.7	------------nnnnnn-G--A------X	
NC_000014.7	TTTTGCTGTCTTGCCTCAAACTGCAAAAG	31.7	X------T--C-nnnnnn-----------	
NC_000014.7	TTTTCAGTTTTGTGAGAAACTGCAAAA	31.7	?X---T------nnnnnn----------X	
NC_000014.7	TTTTGCAGTTTCTCACAAAACTGAAAA	31.7	X-----------nnnnnn------A---X?	
NC_000014.7	CTTTTGTTGTTGCACCTCAAACTGCAAAAG	31.7	------TT---Gnnnnnn-----------	
NC_000014.7	CTTTTGCAGTTTGAGGTGCAACAACAAAAG	31.7	------------nnnnnnC---AA------	
NC_000014.7	CTTTTGCAGTTTGAGATGTGACAGCAAAA	31.7	------------nnnnnnTG--A------X	
NC_000014.7	TTTTGCTGTCACATCTCAAACTGCAAAAG	31.7	X------T--CAnnnnnn-----------	
NC_000014.7	TTATTCACTTTTTGAGTAAACTGCAAAAG	31.7	X--A-T--C---nnnnnn-----------	
NC_000014.7	CTTTTGCAGTTTACTCAAAAAGTGAATAA	31.7	------------nnnnnn---G--A-T--X	
NC_000014.7	CTTTTGCAGTTTAAGATATGACAGCAAAA	31.7	------------nnnnnnTG--A------X	
NC_000014.7	TTTTGCTGTCATATCTTAAACTGCAAAAG	31.7	X------T--CAnnnnnn-----------	
NC_000021.7	TTTTGCTGTTTCAGCTTAAACTGCAAAAG	34.9	X------T----nnnnnn-----------	6 For.
NC_000021.7	CTTTTGCAGTTTAAGCTGAAACAGCAAAA	34.9	------------nnnnnn----A------X	6 Rev.
NC_000021.7	TTTTGCAGTTTGAGGTGCAACGGCAAAA	34.9	X-----------nnnnnnC---G------X	
NC_000021.7	TTTTGCCGTTGCACCTCAAACTGCAAAA	34.9	X------C---Gnnnnnn----------X	
NC_000021.7	TTTTGCAGTTTTAGTAGAGACTGC	34.9	X-----------nnnnnn-G-----X????	
NC_000021.7	GCAGTCTCTACTAAAACTGCAAAA	34.9	????X-----C-nnnnnn----------X	
NC_000021.7	GCAGTATTATTTGAAACTGCAAAA	34.9	????X-----A-nnnnnn----------X	
NC_000015.8	TTTTGCAGTTTGGCAGCAAACTGCAAA	34.9	X-----------nnnnnn---------X?	
NC_000015.8	TTTGCAGTTTGCTGCCAAACTGCAAAA	34.9	?X----------nnnnnn----------X	
NC_000015.8	CTTTTGCAGTTTGAGGTGCAACAGCAAAA	31.7	------------nnnnnnC---A------X	
NC_000015.8	TTTTGCTGTTGCACCTCAAACTGCAAAAG	31.7	X------T---Gnnnnnn-----------	
NC_000015.8	TTGCTGTTGCACCTCAAACTGCAAAAG	31.7	??X----T---Gnnnnnn-----------	
NC_000015.8	CTTTTGCAGTTTGAGGTGCAACAGCAA	31.7	------------nnnnnnC---A----X??	
NC_000015.8	CTTTTGCAGTTTAAGGTGAAAATGC	31.7	------------nnnnnn---A---X????	
NC_000015.8	GCATTTTCACCTTAAACTGCAAAAG	31.7	????X---T---nnnnnn-----------	
NC_000015.8	CTTTTGCAGTTTGAGGTATGACAGCAAAA	31.7	------------nnnnnnTG--A------X	
NC_000015.8	TTTTGCTGTCATACCTCAAACTGCAAAAG	31.7	X------T--CAnnnnnn-----------	
NC_000015.8	TTTTGCAGTTTACTCAAAAAGTGAACAA	31.7	X-----------nnnnnn---G--A-C--X	
NC_000015.8	TTGTTCACTTTTTGAGTAAACTGCAAAA	31.7	X--G-T--C---nnnnnn----------X	
NC_000008.9	TTTTGCTGTTTCACCTAAAACTGCAAAAG	34.9	X------T----nnnnnn-----------	7 For.
NC_000008.9	CTTTTGCAGTTTTAGGTGAAACAGCAAAA	34.9	------------nnnnnn----A------X	7 Rev.
NC_000008.9	TTTTGCTGTTGTATCTCAAACTGCAAAAG	31.7	X------T---Gnnnnnn-----------	
NC_000008.9	CTTTTGCAGTTTGAGATACAACAGCAAAA	31.7	------------nnnnnnC---A------X	
NC_000008.9	TTTGTAGTTTTGCATCTAACTGCAAAAG	31.7	?X----T-----nnnnnnT----------	
NC_000008.9	CTTTTGCAGTTAGATGCAAAACTACAAA	31.7	-----------Annnnnn-----A----X?	
NC_000008.9	CTTTTGCAGTTTGGGGTGCAACAGTAAAA	31.7	------------nnnnnnC---A-T----X	
NC_000008.9	TTTCGCTGTTGCACTTCAAACTGCAAAAG	31.7	X---C--T---Gnnnnnn-----------	
NC_000008.9	CTTTTGCAGTTTGAAGTGCAACAGCGAAA	31.7	------------nnnnnnC---A--G---X	
NC_000008.9	TTTTGCTGTCACACTTCAAACTGCAAAAG	31.7	X------T--CAnnnnnn-----------	
NC_000008.9	CTTTTGCAGTTTGAAGTGTGACAGCAAAA	31.7	------------nnnnnnTG--A------X	
NC_000008.9	TTTTGCTGTGATACCTCAAACTGCAAAAG	31.7	X------T--GAnnnnnn-----------	
NC_000008.9	CTTTTGCAGTTTGAGGTATCACAGCAAAA	31.7	------------nnnnnnTC--A------X	
NC_000008.9	CTTTTGCAGTTTGAGGTGTGACAGCAAAA	31.7	------------nnnnnnTG--A------X	
NC_000008.9	TTTTGCTGTCACACCTCAAACTGCAAAAG	31.7	X------T--CAnnnnnn-----------	
NC_000008.9	CTTTTGCAGTTTGAGGTGTGACAGCAAAA	31.7	------------nnnnnnTG--A------X	
NC_000008.9	TTTTGCTGTCACACCTCAAACTGCAAAAG	31.7	X------T--CAnnnnnn-----------	
NC_000008.9	TTTTGCTGTCATACCTCAAACTGCAAAAG	31.7	X------T--CAnnnnnn-----------	
NC_000008.9	CTTTTGCAGTTTGAGGTATGACAGCAAAA	31.7	------------nnnnnnTG--A------X	
NC_000008.9	TTTTGCTGTCACACTGCAAACTGCAAAAG	31.7	X------T--CAnnnnnn-----------	
NC_000008.9	CTTTTGCAGTTTGCAGTGTGACAGCAAAA	31.7	------------nnnnnnTG--A------X	
NC_000008.9	TTTTGCAGTTTGGAAAGAAACT	31.7	X-----------nnnnnn-----X??????	
NC_000008.9	AGTTTCTTTCCAAACTGCAAAA	31.7	??????X-----nnnnnn----------X	
NC_000008.9	TTTTGTATTTTTGAGGGAAACTGCAA	31.7	X-----T-T---nnnnnn--------X??	
NC_000008.9	TTGCAGTTTCCCTCAAAAATACAAAA	31.7	??X---------nnnnnn---A-A-----X	
NC_000008.9	CTTTTGCAGTTTTAAAAACACACAAAAAG	31.7	------------nnnnnn-C--ACA-----	
NC_000008.9	CTTTTTGTGTGTTTTTTAAAACTGCAAAAG	31.7	-----TGT--G-nnnnnn-----------	
NC_000017.9	CTTTTGCTGTTGCACCTCAAACTGCAAAAG	33.3	-------T---Gnnnnnn-----------	3 For.
NC_000017.9	CTTTTGCAGTTTGAGGTGCAACAGCAAAAG	33.3	------------nnnnnnC---A-------	3 Rev.
NC_000017.9	CTTTTGCAGTTTGAGGTCCAACAGCAAAA	31.7	------------nnnnnnC---A------X	
NC_000017.9	TTTTGCTGTTGGACCTCAAACTGCAAAAG	31.7	X------T---Gnnnnnn-----------	
NC_000017.9	TTTTGCTGTTGCACCTCAAACTGCAAAAG	31.7	X------T---Gnnnnnn-----------	
NC_000017.9	CTTTTGCAGTTTGAGGTGCAACAGCAAAA	31.7	------------nnnnnnC---A------X	
NC_000017.9	CTTTTGCAGTTTGAGGTGTGACAGCAAAA	31.7	------------nnnnnnTG--A------X	
NC_000017.9	TTTTGCTGTCACACCTCAAACTGCAAAAG	31.7	X------T--CAnnnnnn-----------	
NC_000017.9	CTTTTGCAGTTTGAGGTGTGACAGCAAAA	31.7	------------nnnnnnTG--A------X	
NC_000017.9	TTTTGCTGTCACACCTCAAACTGCAAAAG	31.7	X------T--CAnnnnnn-----------	
NC_000017.9	CTTTTGCAGTTTGAATTGCGACAGCAAAA	31.7	------------nnnnnnCG--A------X	
NC_000017.9	TTTTGCTGTCGCAATTCAAACTGCAAAAG	31.7	X------T--CGnnnnnn-----------	

NC_000017.9	CTTTTGCAGTTCAAGGTGCAACAGCAAAA	31.7	-----------CnnnnnnC---A------X
NC_000017.9	TTTTGCTGTTGCACCTTGAACTGCAAAAG	31.7	X------T---GnnnnnnG-----------
NC_000023.9	CTTTTGCAGTTAGAGGTGCAACTGCAAAA	31.7	-----------AnnnnnnC----------X
NC_000023.9	TTTTGCAGTTGCACCTCTAACTGCAAAAG	31.7	X----------GnnnnnnT-----------
NC_000023.9	TTTTCAGTTTCTGAAAAAACTGCAAAA	31.7	?X---T------nnnnnn-----------X
NC_000023.9	TTTTGCAGTTTTTTCAGAAACTGAAAA	31.7	X-----------nnnnnn------A---X?
NC_000023.9	TTTTGCAGTTTGATTATAATCTGCAGAA	31.7	X-----------nnnnnn--T-----G--X
NC_000023.9	TTCTGCAGATTATAATCAAACTGCAAAA	31.7	X--C-----A--nnnnnn-----------X
NC_000023.9	TTTTGCTGTCACATCTGAAACTGCAAAAG	31.7	X------T--CAnnnnnn------------
NC_000023.9	CTTTTGCAGTTTCAGATGTGACAGCAAAA	31.7	------------nnnnnnTG--A------X
NC_000023.9	CAGTTTCAAGTGAAACTGCAAAA	31.7	?????X------nnnnnn-----------X
NC_000023.9	TTTTGCAGTTTCACTTGAAACTG	31.7	X-----------nnnnnn------X?????
NC_000023.9	CTTTTGCAGTTTGAGATGTGACAGCAAAA	31.7	------------nnnnnnTG--A------X
NC_000023.9	TTTTGCTGTCACATCTCAAACTGCAAAAG	31.7	X------T--CAnnnnnn------------
NC_000023.9	GTTTAGAGTTAAACTGCAAAAG	31.7	???????X----nnnnnn------------
NC_000023.9	CTTTTGCAGTTTTGGAACAAAGTG	31.7	------------nnnnnn---G--X?????
NC_000023.9	AGTTTCTTAACAAACTGCAAAA	31.7	??????X-----nnnnnn-----------X
NC_000023.9	TTTTGCAGTTTGTTAAGAAACT	31.7	X-----------nnnnnn-----X??????
NC_000020.9	TTTTGCTGTTGTACCTCAAACTGCAAAAG	31.7	X------T---Gnnnnnn------------
NC_000020.9	CTTTTGCAGTTTGAGGTACAACAGCAAAA	31.7	------------nnnnnnC---A------X
NC_000020.9	CTTTTGCAGTTTGAGGTATGACAGCAAAA	31.7	------------nnnnnnTG--A------X
NC_000020.9	TTTTGCTGTCATACCTCAAACTGCAAAAG	31.7	X------T--CAnnnnnn------------
NC_000020.9	TTTTACTGTTGCACCTCAAACTGCAAAAG	31.7	X----A-T---Gnnnnnn------------
NC_000020.9	CTTTTGCAGTTTGAGGTGCAACAGTAAAA	31.7	------------nnnnnnC---A-T----X
NC_000020.9	CTTTTGCAGTTTGAGGTACGACAGCAAAA	31.7	------------nnnnnnCG--A------X
NC_000020.9	TTTTGCTGTCGTACCTCAAACTGCAAAAG	31.7	X------T--CGnnnnnn------------
NC_000020.9	TTTTGCTGCTGCACCTCAAACTGCAAAAG	31.7	X------T-C-Gnnnnnn------------
NC_000020.9	CTTTTGCAGTTTGAGGTGCAGCAGCAAAA	31.7	------------nnnnnnC-G-A------X
NC_000020.9	TTTTGCAGTTTTTAAAAACACTGCA	31.7	X-----------nnnnnn-C------X???
NC_000020.9	TGCAGTGTTTTTAAAAACTGCAAAA	31.7	???X------G-nnnnnn-----------X
NC_000020.9	GCAGCTTTACTTAAAACTGCAAAA	31.7	????X----C--nnnnnn-----------X
NC_000020.9	TTTTGCAGTTTTAAGTAAAGCTGC	31.7	X-----------nnnnnn--G----X????
NC_000012.10	CTTTTGCAGTTTGAGATGCAACAGCAAAA	31.7	------------nnnnnnC---A------X
NC_000012.10	TTTTGCTGTTGCATCTCAAACTGCAAAAG	31.7	X------T---Gnnnnnn------------
NC_000012.10	TTTTGCTGTTGCATCTAAAACTGCAAAAG	31.7	X------T---Gnnnnnn------------
NC_000012.10	CTTTTGCAGTTTTAGATGCAACAGCAAAA	31.7	------------nnnnnnC---A------X
NC_000012.10	TTTTGCAGTTTAAAGGGCAACAGCAAAA	31.7	X-----------nnnnnnC---A------X
NC_000012.10	TTTTGCTGTTGCCCTTTAAACTGCAAAA	31.7	X------T---Gnnnnnn-----------X
NC_000012.10	TTTTGCTGTCACACCTTAAACTGCAAAAG	31.7	X------T--CAnnnnnn------------
NC_000012.10	CTTTTGCAGTTTAAGGTGTGACAGCAAAA	31.7	------------nnnnnnTG--A------X
NC_000012.10	TTTTGCTGTCATACCCCAAACTGCAAAAG	31.7	X------T--CAnnnnnn------------
NC_000012.10	TTTTGCTGTCATACTTCAAACTGCAAAAG	31.7	X------T--CAnnnnnn------------
NC_000012.10	CTTTTGCAGTTTGAAGTATGACAGCAAAA	31.7	------------nnnnnnTG--A------X
NC_000012.10	CAGTTTCCTCAAAAACTGCAAAA	31.7	?????X------nnnnnn-----------X
NC_000012.10	TTTTGCAGTTTTTGAGGAAACTG	31.7	X-----------nnnnnn------X?????
NC_000012.10	GTTTTTGAAGAAACTGCAAAAG	31.7	???????X----nnnnnn------------
NC_000012.10	TGCAATGTTTTGCTAAACTGCAAAAG	31.7	???X----A-G-nnnnnn------------
NC_000012.10	TTTTGCAGTTTCTTATAAAACT	31.7	X-----------nnnnnn-----X??????
NC_000012.10	AGTTTTATAAGAAACTGCAAAA	31.7	??????X-----nnnnnn-----------X
NC_000012.10	CTTTTGCAGTTTTGTTTAAAAC	31.7	------------nnnnnn----X???????
NC_000012.10	GTTTTAAACAAAACTGCAAAAG	31.7	???????X----nnnnnn------------
NC_000012.10	TTTTGCTGATGTACCTCAAACTGCAAAA	31.7	X------T-A-Gnnnnnn-----------X
NC_000012.10	TTTTGCAGTTTGAGGTACATCAGCAAAA	31.7	X-----------nnnnnnC-T-A------X
NC_000012.10	TTTTGCAGTTTGAGGTAGGACAGCAAAA	31.7	X-----------nnnnnnGG--A------X
NC_000012.10	TTTTGCTGTCCTACCTCAAACTGCAAAA	31.7	X------T--CCnnnnnn-----------X
NC_000012.10	TTTTGCAGTTTAATGTGCAGCAGCAAAA	31.7	X-----------nnnnnnC-G-A------X
NC_000012.10	TTTTGCTGCTGCACATTAAACTGCAAAA	31.7	X------T-C-Gnnnnnn-----------X
NC_000012.10	CTTTTGCAGTTTGAGGTGCAACAACAAA	31.7	------------nnnnnnC---AA----X?
NC_000012.10	TTTGTTGTTGCACCTCAAACTGCAAAAG	31.7	?X----TT---Gnnnnnn------------
NC_000012.10	TTTTGCAGTTTCTTACAAAACT	31.7	X-----------nnnnnn-----X??????
NC_000012.10	AGTTTTGTAAGAAACTGCAAAA	31.7	??????X-----nnnnnn-----------X
NC_000011.8	CTTTTGCAGTTTGAGGTGCAACAGCAAAA	31.7	------------nnnnnnC---A------X
NC_000011.8	TTTTGCTGTTGCACCTCAAACTGCAAAAG	31.7	X------T---Gnnnnnn------------
NC_000011.8	TTTTGCAGTTTGGAGGAAAAATGCAGAA	31.7	X-----------nnnnnn---A----G--X
NC_000011.8	TTCTGCATTTTTCCTCCAAACTGCAAAA	31.7	X--C----T---nnnnnn-----------X
NC_000011.8	CTTTTGCAGTTTGAGGTGTGACAGCAAAA	31.7	------------nnnnnnTG--A------X
NC_000011.8	TTTTGCTGTCACACCTCAAACTGCAAAAG	31.7	X------T--CAnnnnnn------------
NC_000011.8	TTTTGCTGTGGCACCCCAAACTGCAAAAG	31.7	X------T--GGnnnnnn------------
NC_000011.8	CTTTTGCAGTTTGGGGTGCCACAGCAAAA	31.7	------------nnnnnnCC--A------X
NC_000011.8	TGTAGTTTGGTAGAAAACTGCAAAA	31.7	???X--T-----nnnnnn-----------X
NC_000011.8	TTTTGCAGTTTTCTACCAAACTACA	31.7	X-----------nnnnnn-----A--X???
NC_000011.8	TTTTGCTGTCGCACCTCAAACTGCAAAAG	31.7	X------T--CGnnnnnn------------
NC_000011.8	TTTTGCTGTCGTACCTCAAACTGCAAAAG	31.7	X------T--CGnnnnnn------------

NC_000011.8	CTTTTGCAGTTTGAGGTACGACAGCAAAA	31.7	------------nnnnnnCG--A------X
NC_000011.8	TTTTGCTGTCACATCTCAAACTGCAAAAG	31.7	X------T--CAnnnnnn------------
NC_000011.8	TTTTGCAGTTTCTTACAAAACT	31.7	X-----------nnnnnn-----X??????
NC_000011.8	AGTTTTGTAAGAAACTGCAAAA	31.7	??????X-----nnnnnn-----------X
NC_000011.8	GTTTTTGTAGAAACTGCAAAAG	31.7	???????X----nnnnnn------------
NC_000011.8	CTTTTGCAGTTTCTACAAAAAC	31.7	------------nnnnnn----X???????
NC_000011.8	CTTTTGCAGTTTCTACAAAAAC	31.7	------------nnnnnn----X???????
NC_000011.8	GTTTTTGTAGAAACTGCAAAAG	31.7	???????X----nnnnnn------------
NC_000011.8	GTTTTTGTAGAAACTGCAAAAG	31.7	???????X----nnnnnn------------
NC_000011.8	CTTTTGCAGTTTCTACAAAAAC	31.7	------------nnnnnn----X???????
NC_000011.8	GTTTTTGTAGAAACTGCAAAAG	31.7	???????X----nnnnnn------------
NC_000011.8	CTTTTGCAGTTTCTACAAAAAC	31.7	------------nnnnnn----X???????
NC_000011.8	GTTTTTGTAGAAACTGCAAAAG	31.7	???????X----nnnnnn------------
NC_000011.8	CTTTTGCAGTTTCTACAAAAAC	31.7	------------nnnnnn----X???????
NC_000011.8	GTTTTTGTACAAACTGCAAAAG	31.7	???????X----nnnnnn------------
NC_000011.8	CTTTTGCAGTTTGTACAAAAAC	31.7	------------nnnnnn----X???????
NC_000011.8	GTTTTTGTAGAAACTGCAAAAG	31.7	???????X----nnnnnn------------
NC_000011.8	CTTTTGCAGTTTCTACAAAAAC	31.7	------------nnnnnn----X???????
NC_000011.8	GTTTTTGTAGAAACTGCAAAAG	31.7	???????X----nnnnnn------------
NC_000011.8	CTTTTGCAGTTTCTACAAAAAC	31.7	------------nnnnnn----X???????
NC_000011.8	AGTTTTACAAGAAACTGCAAAA	31.7	??????X-----nnnnnn-----------X
NC_000011.8	TTTTGCAGTTTCTTGTAAAACT	31.7	X-----------nnnnnn-----X??????
NC_000011.8	TTTTGCTGTCATAACTCAAACTGCAAAA	31.7	X------T--CAnnnnnn-----------X
NC_000011.8	TTTTGCAGTTTGAGTTATGACAGCAAAA	31.7	X-----------nnnnnnTG--A------X
NC_000010.9	CTTTTGCAGTTTGAAGTGCAACAGCAAAA	31.7	------------nnnnnnC---A------X
NC_000010.9	TTTTGCTGTTGCACTTCAAACTGCAAAAG	31.7	X------T---Gnnnnnn------------
NC_000010.9	CTTTTGCAGTTTGAGGTGCAACAGCAAAA	31.7	------------nnnnnnC---A------X
NC_000010.9	TTTTGCTGTTGCACCTCAAACTGCAAAAG	31.7	X------T---Gnnnnnn------------
NC_000010.9	CTTTTGCAGTTTGAGATATAACAGCAAAA	31.7	------------nnnnnnT---A------X
NC_000010.9	TTTTGCTGTTATATCTCAAACTGCAAAAG	31.7	X------T---Annnnnn------------
NC_000010.9	TTTTGCTGTTGCACCTCAAACTGCAAAAG	31.7	X------T---Gnnnnnn------------
NC_000010.9	CTTTTGCAGTTTGAGGTGCAACAGCAAAA	31.7	------------nnnnnnC---A------X
NC_000010.9	TTTTGCTTTTGTACCTCAAACTGCAAAAG	31.7	X------TT--Gnnnnnn------------
NC_000010.9	CTTTTGCAGTTTGAGGTACAAAAGCAAAA	31.7	------------nnnnnnC--AA------X
NC_000010.9	CTTTTGCAGTTTCAGGTGTGACAGCAAAA	31.7	------------nnnnnnTG--A------X
NC_000010.9	TTTTGCTGTCACACCTGAAACTGCAAAAG	31.7	X------T--CAnnnnnn------------
NC_000010.9	TTTTGCAGTTTGTTGTAAAACT	31.7	X-----------nnnnnn-----X??????
NC_000010.9	AGTTTTACAACAAACTGCAAAA	31.7	??????X-----nnnnnn-----------X
NC_000010.9	TTTTGCTGTCCAACCTCAAACTGCAAAA	31.7	X------T--CCnnnnnn-----------X
NC_000009.10	TTTTGCTGTTGCACGTCAAACTGCAAAAG	31.7	X------T---Gnnnnnn------------
NC_000009.10	CTTTTGCAGTTTGACGTGCAACAGCAAAA	31.7	------------nnnnnnC---A------X
NC_000009.10	TTTTGCTGTTGCACCTCAAACTGCAAAAG	31.7	X------T---Gnnnnnn------------
NC_000009.10	CTTTTGCAGTTTGAGGTGCAACAGCAAAA	31.7	------------nnnnnnC---A------X
NC_000009.10	TTTTGCAGTTTGGTATGCAACAGCAAAA	31.7	X-----------nnnnnnC---A------X
NC_000009.10	TTTTGCTGTTGCATACCAAACTGCAAAA	31.7	X------T---Gnnnnnn-----------X
NC_000009.10	TTGCATTTTCCCCTACAACTGCAAAAG	31.7	??X-----T---nnnnnnC-----------
NC_000009.10	CTTTTGCAGTTGTAGGGGAAAATGCAA	31.7	-----------Gnnnnnn---A-----X??
NC_000009.10	TTCTGCTGTTGCACCTCAAACTGCAAAAG	31.7	X--C---T---Gnnnnnn------------
NC_000009.10	CTTTTGCAGTTTGAGGTGCAACAGCAGAA	31.7	------------nnnnnnC---A---G--X
NC_000009.10	CTTTTGCAGTTTGAGGTACAACAGTAAAA	31.7	------------nnnnnnC---A-T----X
NC_000009.10	TTTTACTGTTGTACCTCAAACTGCAAAAG	31.7	X----A-T---Gnnnnnn------------
NC_000009.10	CTTTTGCAGTTTGAGGTGCAAAAGCAAAA	31.7	------------nnnnnnC--AA------X
NC_000009.10	TTTTGCTTTTGCACCTCAAACTGCAAAAG	31.7	X------TT--Gnnnnnn------------
NC_000009.10	TTTCTGTTTAAATAAAAACTGCAAAAG	31.7	??X--T-T----nnnnnn------------
NC_000009.10	CTTTTGCAGTTTTTATTTAAACAGAAA	31.7	------------nnnnnn----A-A--X??
NC_000009.10	CTTTTGCAGTTTGAAGTACAACAGAAAAA	31.7	------------nnnnnnC---A-A----X
NC_000009.10	TTTTTCTGTTGTACTTCAAACTGCAAAAG	31.7	X----T-T---Gnnnnnn------------
NC_000009.10	TTTTGCAGTTTACTAAAAAAGTGAACAA	31.7	X-----------nnnnnn---G--A-C--X
NC_000009.10	TTGTTCACTTTTTTAGTAAACTGCAAAA	31.7	X--G-T--C---nnnnnn-----------X
NC_000009.10	AGTTTCACCAGAAACTGCAAAA	31.7	??????X-----nnnnnn-----------X
NC_000009.10	TTTTGCAGTTTCTGGTGAAACT	31.7	X-----------nnnnnn-----X??????
NC_000009.10	CTTTTGCAGTTGGAGATGCAACAGCAAGAG	31.7	-----------GnnnnnnC---A----G--
NC_000009.10	CTCTTGCTGTTGCATCTCCAACTGCAAAAG	31.7	--C----T---GnnnnnnC-----------
NC_000009.10	AGTTTTACAAGAAACTGCAAAA	31.7	??????X-----nnnnnn-----------X
NC_000009.10	TTTTGCAGTTTCTTGTAAAACT	31.7	X-----------nnnnnn-----X??????
NC_000009.10	TTACAGTGTCACAGGAAACTGCAAAA	31.7	??X--A----G-nnnnnn-----------X
NC_000009.10	TTTTGCAGTTTCCTGTGACACTGTAA	31.7	X-----------nnnnnn-C----T--X??
NC_000007.12	TTTTGCTGTTGCATCTAAAACTGCAAAAG	31.7	X------T---Gnnnnnn------------
NC_000007.12	CTTTTGCAGTTTTAGATGCAACAGCAAAA	31.7	------------nnnnnnC---A------X
NC_000007.12	TTTTGCTGTTGCATCTAAAACTGCAAAAG	31.7	X------T---Gnnnnnn------------
NC_000007.12	CTTTTGCAGTTTTAGATGCAACAGCAAAA	31.7	------------nnnnnnC---A------X
NC_000007.12	TTTTGCTGTTGCACCCCAAACTGCAAAAG	31.7	X------T---Gnnnnnn------------
NC_000007.12	CTTTTGCAGTTTGGGGTGCAACAGCAAAA	31.7	------------nnnnnnC---A------X

NC_000007.12	CTTTTGCAGTTTGAGGTGCAACAGCAAA	31.7	------------nnnnnnC---A-----X?
NC_000007.12	TTTGCTGTTGCACCTCAAACTGCAAAAG	31.7	?X-----T---Gnnnnnn------------
NC_000007.12	CAGTTTTTTAAAAAACTGCAAAAG	31.7	?????X------nnnnnn------------
NC_000007.12	CTTTTGCAGTTTTTTAAAAAACTG	31.7	------------nnnnnn------X?????
NC_000007.12	CTTTTGCAGTTTGTTCTGAAACTACA	31.7	------------nnnnnn-----A--X???
NC_000007.12	TGTAGTTTCAGAACAAACTGCAAAAG	31.7	???X--T-----nnnnnn------------
NC_000007.12	TTGCTGTTGCACCTCAAACTGCAAAAG	31.7	??X----T---Gnnnnnn------------
NC_000007.12	CTTTTGCAGTTTGAGGTGCAACAGCAA	31.7	------------nnnnnnC---A----X??
NC_000007.12	TTTTGCAGTTTCTTATAAAACTG	31.7	X-----------nnnnnn------X?????
NC_000007.12	CAGTTTTATAAGAAACTGCAAAA	31.7	?????X------nnnnnn-----------X
NC_000007.12	TTTTGCTGTCACATCTCAAACTGCAAAAG	31.7	X------T--CAnnnnnn------------
NC_000007.12	CTTTTGCAGTTTGAGATGTGACAGCAAAA	31.7	------------nnnnnnTG--A------X
NC_000007.12	TTGCTGTTGTACCTCAAACTGCAAAAG	31.7	??X----T---Gnnnnnn------------
NC_000007.12	CTTTTGCAGTTTGAGGTACAACAGCAA	31.7	------------nnnnnnC---A----X??
NC_000007.12	TTTTGCTGTTGGACCTCAGACTGCAAAAG	31.7	X------T---Gnnnnnn-G----------
NC_000007.12	CTTTTGCAGTTTGAAATGTGACAGCAAAA	31.7	------------nnnnnnTG--A------X
NC_000007.12	TTTTGCTGTCACATTTCAAACTGCAAAAG	31.7	X------T--CAnnnnnn------------
NC_000007.12	TTTTGCTGCTGCACCTTAAACTGCAAAAG	31.7	X------T-C-Gnnnnnn------------
NC_000007.12	CTTTTGCAGTTTAAGGTGCAGCAGCAAAA	31.7	------------nnnnnnC-G-A------X
NC_000007.12	AGTTTATTAAGAAACTGCAAAA	31.7	??????X-----nnnnnn-----------X
NC_000007.12	TTTTGCAGTTTCTTAATAAACT	31.7	X-----------nnnnnn-----X??????
NC_000007.12	GTTTTGGTAGAAACTGCAAAAG	31.7	???????X----nnnnnn------------
NC_000007.12	CTTTTGCAGTTTCTACCAAAAC	31.7	------------nnnnnn----X???????
NC_000007.12	GTTTTTGTAGAAACTGCAAAAG	31.7	???????X----nnnnnn------------
NC_000007.12	CTTTTGCAGTTTCTACAAAAAC	31.7	------------nnnnnn----X???????
NC_000007.12	GTTTTTGTAGAAACTGCAAAAG	31.7	???????X----nnnnnn------------
NC_000007.12	CTTTTGCAGTTTCTACAAAAAC	31.7	------------nnnnnn----X???????
NC_000007.12	GTTTTTATAGAAACTGCAAAAG	31.7	???????X----nnnnnn------------
NC_000007.12	CTTTTGCAGTTTCTATAAAAAC	31.7	------------nnnnnn----X???????
NC_000007.12	GTTTTTTTAGAAACTGCAAAAG	31.7	???????X----nnnnnn------------
NC_000007.12	CTTTTGCAGTTTCTAAAAAAAC	31.7	------------nnnnnn----X???????
NC_000007.12	GTTTTTGTAGAAACTGCAAAAG	31.7	???????X----nnnnnn------------
NC_000007.12	CTTTTGCAGTTTCTACAAAAAC	31.7	------------nnnnnn----X???????
NC_000007.12	GTTTTTGTAGAAACTGCAAAAG	31.7	???????X----nnnnnn------------
NC_000007.12	CTTTTGCAGTTTCTACAAAAAC	31.7	------------nnnnnn----X???????
NC_000007.12	GTTTTTGAAGAAACTGCAAAAG	31.7	???????X----nnnnnn------------
NC_000007.12	CTTTTGCAGTTTCTTCAAAAAC	31.7	------------nnnnnn----X???????
NC_000007.12	GTTTTTGTAGAAACTGCAAAAG	31.7	???????X----nnnnnn------------
NC_000007.12	CTTTTGCAGTTTCTACAAAAAC	31.7	------------nnnnnn----X???????
NC_000007.12	GTTTTTGTAGAAACTGCAAAAG	31.7	???????X----nnnnnn------------
NC_000007.12	CTTTTGCAGTTTCTACAAAAAC	31.7	------------nnnnnn----X???????
NC_000007.12	TTTTGCTGTCACACCTCAAACTGCAAAA	31.7	X------T--CAnnnnnn-----------X
NC_000007.12	TTTTGCAGTTTGAGGTGTGACAGCAAAA	31.7	X-----------nnnnnnTG--A------X
NC_000007.12	TTTTGCAGTTTCTTACAAAACT	31.7	X-----------nnnnnn-----X??????
NC_000007.12	AGTTTTGTAAGAAACTGCAAAA	31.7	??????X-----nnnnnn-----------X
NC_000007.12	CTTTTGCAGTTGTCAGGTCCACTGCAAA	31.7	-----------GnnnnnnCC--------X?
NC_000006.10	CTTTTGCAGTTTGAGATACAACAGCAAAA	31.7	------------nnnnnnC---A------X
NC_000006.10	TTTTGCTGTTGTATCTCAAACTGCAAAAG	31.7	X------T---Gnnnnnn------------
NC_000006.10	TTTTGCTGTCTCACCCCAAACTGCAAAA	31.7	X------T--C-nnnnnn-----------X
NC_000006.10	TTTTGCAGTTTGGGGTGAGACAGCAAAA	31.7	X-----------nnnnnn-G--A------X
NC_000006.10	TTTTGCAGTTTCTTATGAAACT	31.7	X-----------nnnnnn-----X??????
NC_000006.10	AGTTTCATAAGAAACTGCAAAA	31.7	??????X-----nnnnnn-----------X
NC_000006.10	CTTTTGCAGTTTCTACAAAAAC	31.7	------------nnnnnn----X???????
NC_000006.10	GTTTTTGTAGAAACTGCAAAAG	31.7	???????X----nnnnnn------------
NC_000006.10	TTGTTCACTTTTTGAGTAAACTGCAAAA	31.7	X--G-T--C---nnnnnn-----------X
NC_000006.10	TTTTGCAGTTTACTCAAAAAGTGAACAA	31.7	X-----------nnnnnn---G--A-C--X
NC_000006.10	TTTTCAGATTTTCTAGAAACTGCAAA	31.7	?X---T---A--nnnnnn----------X?
NC_000006.10	TTTGCAGTTTCTAGAAAATCTGAAAA	31.7	?X----------nnnnnn--T---A---X?
NC_000005.8	TTTTGCTGTCTCATGTCAAACTGCAAAAG	31.7	X------T--C-nnnnnn------------
NC_000005.8	CTTTTGCAGTTTGACATGAGACAGCAAAA	31.7	------------nnnnnn-G--A------X
NC_000005.8	CTTTTGCAGTTTGAGGTACAACAGCAAAA	31.7	------------nnnnnnC---A------X
NC_000005.8	TTTTGCTGTTGTACCTCAAACTGCAAAAG	31.7	X------T---Gnnnnnn------------
NC_000005.8	TTTTGCAGTTTGAAGTGCAACAGCAAAA	31.7	X-----------nnnnnnC---A------X
NC_000005.8	TTTTGCTGTTGCACTTCAAACTGCAAAA	31.7	X------T---Gnnnnnn-----------X
NC_000005.8	CTTTTGCAGTTTGAGGTATAACCACAAAA	31.7	------------nnnnnnT---CA-----X
NC_000005.8	TTTTGTGGTTATACCTCAAACTGCAAAAG	31.7	X-----TG---Annnnnn------------
NC_000005.8	CTTTTGCAGTTTGAGATGCAACATCAAAA	31.7	------------nnnnnnC---AT-----X
NC_000005.8	TTTTGATGTTGCATCTCAAACTGCAAAAG	31.7	X-----AT---Gnnnnnn------------
NC_000005.8	CAGTTTTGGGAGAAACTGCAAAA	31.7	?????X------nnnnnn-----------X
NC_000005.8	TTTTGCAGTTTCTCCCAAAACTG	31.7	X-----------nnnnnn------X?????
NC_000005.8	TTTTGCTGTCATATCTCAAACTGCAAAAG	31.7	X------T--CAnnnnnn------------
NC_000005.8	CTTTTGCAGTTTGAGATATGACAGCAAAA	31.7	------------nnnnnnTG--A------X
NC_000005.8	CTTTTGCAGTTTGAAGTCTGACAGCAAAA	31.7	------------nnnnnnTG--A------X

NC_000005.8	TTTTGCTGTCAGACTTCAAACTGCAAAAG	31.7	X------T--CAnnnnnn------------
NC_000005.8	CTTTTGCAGTTTGAAGTGCAACATCAAAA	31.7	------------nnnnnnC---AT-----X
NC_000005.8	TTTTGATGTTGCACTTCAAACTGCAAAAG	31.7	X-----AT---Gnnnnnn------------
NC_000005.8	TTTTGCTGTTGCACCACTAACTGCAAAAG	31.7	X------T---GnnnnnnT-----------
NC_000005.8	CTTTTGCAGTTAGTGGTGCAACAGCAAAA	31.7	-----------AnnnnnnC---A------X
NC_000005.8	TTTTGCTGTCACACCCCAAACTGCAAAAG	31.7	X------T--CAnnnnnn------------
NC_000005.8	CTTTTGCAGTTTGGGGTGTGACAGCAAAA	31.7	------------nnnnnnTG--A------X
NC_000005.8	TTTTGCTGTCCCACCTCAAACTGCAAAA	31.7	X------T--CCnnnnnn-----------X
NC_000005.8	TTTTGCAGTTTGAGGTGGGACAGCAAAA	31.7	X-----------nnnnnnGG--A------X
NC_000005.8	CTTTTGCAGTTTCTACAGAAAC	31.7	------------nnnnnn----X???????
NC_000005.8	GTTTCTGTAGAAACTGCAAAAG	31.7	???????X----nnnnnn------------
NC_000005.8	AGTTTTGTGAGAAACTGCAAAA	31.7	??????X-----nnnnnn-----------X
NC_000005.8	TTTTGCAGTTTCTCACAAAACT	31.7	X-----------nnnnnn-----X??????
NC_000005.8	TTTTGCAGTTTCCTATGAAGCTGAAA	31.7	X-----------nnnnnn--G---A--X??
NC_000005.8	TTTCAGCTTCATAGGAAACTGCAAAA	31.7	??X--T---C--nnnnnn-----------X
NC_000005.8	GTTTTGTACTAAACTGCAAAAG	31.7	???????X----nnnnnn------------
NC_000005.8	CTTTTGCAGTTTAGTACAAAAC	31.7	------------nnnnnn----X???????
NC_000005.8	AGTTTTTTAAGAAACTGCAAAA	31.7	??????X-----nnnnnn-----------X
NC_000005.8	TTTTGCAGTTTCTTAAAAAACT	31.7	X-----------nnnnnn-----X??????
NC_000005.8	AGTTTTTGTGTAAACTGCAAAA	31.7	??????X-----nnnnnn-----------X
NC_000005.8	TTTTGCAGTTTACACAAAAACT	31.7	X-----------nnnnnn-----X??????
NC_000004.10	TTTTGCTGTTGTGCCTCAAACTGCAAAAG	31.7	X------T---Gnnnnnn------------
NC_000004.10	CTTTTGCAGTTTGAGGCACAACAGCAAAA	31.7	------------nnnnnnC---A------X
NC_000004.10	CTTTTGGTGTTACACATCAAACTGCAAAAG	31.7	------GT---Annnnnn------------
NC_000004.10	CTTTTGCAGTTTGATGTGTAACACCAAAAG	31.7	------------nnnnnnT---AC------
NC_000004.10	CTTTTGCAGTTTGAGGTATGACAGCAAAA	31.7	------------nnnnnnTG--A------X
NC_000004.10	TTTTGCTGTCATACCTCAAACTGCAAAAG	31.7	X------T--CAnnnnnn------------
NC_000004.10	CTTTTGCAGTTTGAGGTGCAACAACAAAA	31.7	------------nnnnnnC---AA-----X
NC_000004.10	TTTTGTTGTTGCACCTCAAACTGCAAAAG	31.7	X-----TT---Gnnnnnn------------
NC_000004.10	TTTTGCTGTCGCACTTCAAACTGCAAAAG	31.7	X------T--CGnnnnnn------------
NC_000004.10	CTTTTGCAGTTTGAAGTGCGACAGCAAAA	31.7	------------nnnnnnCG--A------X
NC_000004.10	CTTTTGCAGTTTGAGGTGCAGCAGCAAAA	31.7	------------nnnnnnC-G-A------X
NC_000004.10	TTTTGCTGCTGCACCTCAAACTGCAAAAG	31.7	X------T-C-Gnnnnnn------------
NC_000004.10	CTTTTGCAGTTTAACATGGGACAGCAAAA	31.7	------------nnnnnnGG--A------X
NC_000004.10	TTTTGCTGTCCCATGTTAAACTGCAAAAG	31.7	X------T--CCnnnnnn------------
NC_000004.10	TTTTACTGTTGCACCTCAAACTGCAAAAG	31.7	X----A-T---Gnnnnnn------------
NC_000004.10	CTTTTGCAGTTTGAGGTGCAACAGTAAAA	31.7	------------nnnnnnC---A-T----X
NC_000004.10	CTTTTGCAGTTTGAAGTATGACAGCAAAA	31.7	------------nnnnnnTG--A------X
NC_000004.10	TTTTGCTGTCATACTTCAAACTGCAAAAG	31.7	X------T--CAnnnnnn------------
NC_000004.10	TTTTGCAGTTTTTAAAGGAACTTTAAAA	31.7	X-----------nnnnnnG----TT----X
NC_000004.10	TTTTAAAGTTCCTTTAAAAACTGCAAAA	31.7	X----AA----Cnnnnnn-----------X
NC_000004.10	TTCTGGAGTCTCATTTAAAACTGCAAAA	31.7	X--C--G---C-nnnnnn-----------X
NC_000004.10	TTTTGCAGTTTTAAATGAGACTCCAGAA	31.7	X-----------nnnnnn-G---C--G--X
NC_000004.10	TTTTGCAGTTTCTTACAAAACT	31.7	X-----------nnnnnn-----X??????
NC_000004.10	AGTTTTGTAAGAAACTGCAAAA	31.7	??????X-----nnnnnn-----------X
NC_000004.10	GTTTTTTGTAAAACTGCAAAAG	31.7	???????X----nnnnnn------------
NC_000004.10	CTTTTGCAGTTTTACAAAAAAC	31.7	------------nnnnnn----X???????
NC_000004.10	TTTTGCAGTTTCTTACAAAACT	31.7	X-----------nnnnnn-----X??????
NC_000004.10	AGTTTTGTAAGAAACTGCAAAA	31.7	??????X-----nnnnnn-----------X
NC_000004.10	CTTTTGCAGTTTCTTAAGTAAATTCCAAAG	31.7	------------nnnnnnT--A-T-C----
NC_000004.10	CTTTGGAATTTACTTAAGAAACTGCAAAAG	31.7	----G-A-T--Annnnnn------------
NC_000003.10	TTTTGCTGTTGCACCTCAAACTGCAAAAG	31.7	X------T---Gnnnnnn------------
NC_000003.10	CTTTTGCAGTTTGAGGTGCAACAGCAAAA	31.7	------------nnnnnnC---A------X
NC_000003.10	TTTTGCTGTTGGACCTCAAACTGCAAAAG	31.7	X------T---Gnnnnnn------------
NC_000003.10	CTTTTGCAGTTTGAGGTCCAACAGCAAAA	31.7	------------nnnnnnC---A------X
NC_000003.10	TTTTGCTGTTACACCTGAAACTGCAAAAG	31.7	X------T---Annnnnn------------
NC_000003.10	CTTTTGCAGTTTCAGGTGTAACAGCAAAA	31.7	------------nnnnnnT---A------X
NC_000003.10	TTTTGCAGTTTGAAGTGAGACAGCAAAA	31.7	X-----------nnnnnn-G--A------X
NC_000003.10	TTTTGCTGTCTCACTTCAAACTGCAAAA	31.7	X------T--C-nnnnnn-----------X
NC_000003.10	TTTTGCTGTTGCATCTCAAACTGCAAAA	31.7	X------T---Gnnnnnn-----------X
NC_000003.10	TTTTGCAGTTTGAGATGCAACAGCAAAA	31.7	X-----------nnnnnnC---A------X
NC_000003.10	TTTTGCTGTCACACTTCAAACTGCAAAAG	31.7	X------T--CAnnnnnn------------
NC_000003.10	CTTTTGCAGTTTGAAGTGTGACAGCAAAA	31.7	------------nnnnnnTG--A------X
NC_000003.10	CTTTTGCAGTTTGAGGTACAACAACAAAA	31.7	------------nnnnnnC---AA-----X
NC_000003.10	TTTTGTTGTTGTACCTCAAACTGCAAAAG	31.7	X-----TT---Gnnnnnn------------
NC_000003.10	TTTGGAGTGTAGAAAAAAACTGCAAAA	31.7	?X----G---G-nnnnnn-----------X
NC_000003.10	AGTTTAACGCAAAACTGCAAAAG	31.7	??????X-----nnnnnn------------
NC_000003.10	CTTTTGCAGTTTTGCGTTAAACT	31.7	------------nnnnnn-----X??????
NC_000003.10	TTTTGCTGGTGAACCTCAAACTGCAAAAG	31.7	X------T-G-Gnnnnnn------------
NC_000003.10	CTTTTGCAGTTTGAGGTTCACCAGCAAAA	31.7	------------nnnnnnC-C-A------X
NC_000003.10	TTGTGCTGTTGTACTTCAAACTGCAAAAG	31.7	X--G---T---Gnnnnnn------------
NC_000003.10	CTTTTGCAGTTTGAAGTACAACAGCACAA	31.7	------------nnnnnnC---A---C--X
NC_000003.10	TTTTGCTGTAGCACCTCAAACTGCAAAAG	31.7	X------T--AGnnnnnn------------

NC_000003.10	CTTTTGCAGTTTGAGGTGCTACAGCAAAA	31.7	------------nnnnnnCT--A------X
NC_000003.10	CTTTTGCAGTTTAAGGCGCAGCAGCAAAA	31.7	------------nnnnnnC-G-A------X
NC_000003.10	TTTTGCTGCTGCGCCTTAAACTGCAAAAG	31.7	X------T-C-Gnnnnnn------------
NC_000003.10	TTTGCTGTAGCACCTCAAACTGCAAAAG	31.7	?X-----T--AGnnnnnn------------
NC_000003.10	CTTTTGCAGTTTGAGGTGCTACAGCAAA	31.7	------------nnnnnnCT--A-----X?
NC_000003.10	TTATGCAGTGATAACACAAACTGCAAAA	31.7	X--A------GAnnnnnn-----------X
NC_000003.10	CTTTTGCAGTTTTAGTAGAAAC	31.7	------------nnnnnn----X???????
NC_000003.10	GTTTCTACTAAAACTGCAAAAG	31.7	???????X----nnnnnn------------
NC_000003.10	AGTTTTATTAGAAACTGCAAAA	31.7	??????X-----nnnnnn-----------X
NC_000003.10	TTTTGCAGTTTCTAATAAAACT	31.7	X-----------nnnnnn-----X??????
NC_000003.10	AGTTTCAAAATAAACTGCAAAA	31.7	??????X-----nnnnnn-----------X
NC_000003.10	AGTTTGAAAACAAACTGCAAAA	31.7	??????X-----nnnnnn-----------X
NC_000003.10	TTTTGCAGTTTGTTTTCAAACT	31.7	X-----------nnnnnn-----X??????
NC_000003.10	TTTTGCAGTTTGAGGTGCAACAGGAAAA	31.7	X-----------nnnnnnC---A-G----X
NC_000003.10	TTTTCCTGTTGCACCTCAAACTGCAAAA	31.7	X----C-T---Gnnnnnn-----------X
NC_000002.10	CTTTTGCAGTTTGAGGTGCAACAGCAAAA	31.7	------------nnnnnnC---A------X
NC_000002.10	TTTTGCTGTTGCACCTCAAACTGCAAAAG	31.7	X------T---Gnnnnnn------------
NC_000002.10	TTTTGCAGTTTGAGGTGCAACAGCAAAAG	31.7	X-----------nnnnnnC---A-------
NC_000002.10	CTTTTGCTGTTGCACCTCAAACTGCAAAA	31.7	-------T---Gnnnnnn-----------X
NC_000002.10	CTTTTGCAGTTTGAGGTGCAACAGCAAAA	31.7	------------nnnnnnC---A------X
NC_000002.10	TTTTGCTGTTGCACCTCAAACTGCAAAAG	31.7	X------T---Gnnnnnn------------
NC_000002.10	CTTTTGCAGTTTGAGGTACAACAGCAAAA	31.7	------------nnnnnnC---A------X
NC_000002.10	TTTTGCTGTTGTACCTCAAACTGCAAAAG	31.7	X------T---Gnnnnnn------------
NC_000002.10	TTTTGCTGTTGCATCTGAAACTGCAAAAG	31.7	X------T---Gnnnnnn------------
NC_000002.10	CTTTTGCAGTTTCAGATGCAACAGCAAAA	31.7	------------nnnnnnC---A------X
NC_000002.10	TTTGCTGTTGAATCTCAAACTGCAAAAG	31.7	?X-----T---Gnnnnnn------------
NC_000002.10	CTTTTGCAGTTTGAGATTCAACAGCAAA	31.7	------------nnnnnnC---A-----X?
NC_000002.10	CTTTTGCAGTTTGAGGTGTGACAGCAAAA	31.7	------------nnnnnnTG--A------X
NC_000002.10	TTTTGCTGTCACACCTCAAACTGCAAAAG	31.7	X------T--CAnnnnnn------------
NC_000002.10	CTTTTGCAGTTTGAGGTGCAACAGCACAA	31.7	------------nnnnnnC---A---C--X
NC_000002.10	TTGTGCTGTTGCACCTCAAACTGCAAAAG	31.7	X--G---T---Gnnnnnn------------
NC_000002.10	TTTTGCTGTCGCACCTCAAACTGCAAAAG	31.7	X------T--CGnnnnnn------------
NC_000002.10	TTTTGCTGTTGTGCCTCAGACTGCAAAAG	31.7	X------T---Gnnnnnn-G----------
NC_000002.10	TTTTGCTGTCACATCTCAAACTGCAAAAG	31.7	X------T--CAnnnnnn------------
NC_000002.10	CTTTTGCAGTTTGAGATGTGACAGCAAAA	31.7	------------nnnnnnTG--A------X
NC_000002.10	CTTTTGCAGTTTGAGGTGCACCAGCAAAA	31.7	------------nnnnnnC-C-A------X
NC_000002.10	TTTTGCTGGTGCACCTCAAACTGCAAAAG	31.7	X------T-G-Gnnnnnn------------
NC_000002.10	CTTTTGCAGTTTGAGGTGTGACAGCAAAA	31.7	------------nnnnnnTG--A------X
NC_000002.10	TTTTGCTGTCACACCTCAAACTGCAAAAG	31.7	X------T--CAnnnnnn------------
NC_000002.10	TTTTGCAGTTTGATTAGAGACTTCTAAAG	31.7	X-----------nnnnnn-G---T-T----
NC_000002.10	CTTTAGAAGTCTCTAATCAAACTGCAAAA	31.7	----A-A---C-nnnnnn-----------X
NC_000002.10	CTTTTGCAGTTTGAGGCAAGACAGCA	31.7	------------nnnnnn-G--A---X???
NC_000002.10	TGCTGTCTTGCCTCAAACTGCAAAAG	31.7	???X---T--C-nnnnnn------------
NC_000002.10	AGTTTTCAAGAAAACTGCAAAA	31.7	??????X-----nnnnnn-----------X
NC_000002.10	TTTTGCAGTTTTCTTGAAAACT	31.7	X-----------nnnnnn-----X??????
NC_000002.10	TTTTGCAGTTTGAGGAGCAGCAGCAAAA	31.7	X-----------nnnnnnC-G-A------X
NC_000002.10	TTTTGCTGCTGCTCCTCAAACTGCAAAA	31.7	X------T-C-Gnnnnnn-----------X
NC_000002.10	TTTTGCTGGTGTACCTCAAACTGCAAAA	31.7	X------T-G-Gnnnnnn-----------X
NC_000002.10	TTTTGCAGTTTGAGGTACACCAGCAAAA	31.7	X-----------nnnnnnC-C-A------X
NC_000002.10	CTTTTGCAGTTTGAGGTGCAACACAAAAAG	31.7	------------nnnnnnC---ACA-----
NC_000002.10	CTTTTTGTGTTGCACCTCAAACTGCAAAAG	31.7	-----TGT---Gnnnnnn------------
NC_000001.9	TTTTGCAGTTTGAAGTGCAACAGCAAAAG	31.7	X-----------nnnnnnC---A-------
NC_000001.9	CTTTTGCTGTTGCACTTCAAACTGCAAAA	31.7	-------T---Gnnnnnn-----------X
NC_000001.9	TTTTGCTGTTATACATCAAACTGCAAAAG	31.7	X------T---Annnnnn------------
NC_000001.9	CTTTTGCAGTTTGATGTATAACAGCAAAA	31.7	------------nnnnnnT---A------X
NC_000001.9	CTTTTGCAGTTTGAAGTGCAACAGCAAAA	31.7	------------nnnnnnC---A------X
NC_000001.9	TTTTGCTGTTGCACTTCAAACTGCAAAAG	31.7	X------T---Gnnnnnn------------
NC_000001.9	TTTTGCAGTTTCATGTGCAACAGCAAAA	31.7	X-----------nnnnnnC---A------X
NC_000001.9	TTTTGCTGTTGCACATGAAACTGCAAAA	31.7	X------T---Gnnnnnn-----------X
NC_000001.9	CTTTTGCAGTTTGAGGTACAAAGGCAAAAG	31.7	------------nnnnnnC--AG-------
NC_000001.9	CTTTTGCCTTTGTACCTCAAACTGCAAAAG	31.7	-------CT--Gnnnnnn------------
NC_000001.9	CTTTTGCAGTTTGAAGTACATCAGCAAAA	31.7	------------nnnnnnC-T-A------X
NC_000001.9	TTTTGCTGATGTACTTCAAACTGCAAAAG	31.7	X------T-A-Gnnnnnn------------
NC_000001.9	CTTTTAAAGTTAGTATCTAAACTGCAAAA	31.7	-----AA----Annnnnn-----------X
NC_000001.9	TTTTGCAGTTTAGATACTAACTTTAAAAG	31.7	X-----------nnnnnnT----TT-----
NC_000001.9	TTTTGCTGTCACACCTCAAACTGCAAAAG	31.7	X------T--CAnnnnnn------------
NC_000001.9	CTTTTGCAGTTTGAGGTGTGACAGCAAAA	31.7	------------nnnnnnTG--A------X
NC_000001.9	CTTTTGCAGTTTGAGGTATGACAGCAAAA	31.7	------------nnnnnnTG--A------X
NC_000001.9	TTTTGCTGTCATACCTCAAACTGCAAAAG	31.7	X------T--CAnnnnnn------------
NC_000001.9	CTTTTGCAGTTTGAGGTACAACAACAAAA	31.7	------------nnnnnnC---AA-----X
NC_000001.9	TTTTGTTGTTGTACCTCAAACTGCAAAAG	31.7	X-----TT---Gnnnnnn------------
NC_000001.9	TTTTGCTGTTGCACCTCATACTGCAAAAG	31.7	X------T---Gnnnnnn-T----------
NC_000001.9	TTTTGCTGTCACACTTCAAACTGCAAAAG	31.7	X------T--CAnnnnnn------------

NC_000001.9	CTTTTGCAGTTTGAAGTGTGACAGCAAAA	31.7	------------nnnnnnTG--A------X
NC_000001.9	TTTTGCTGTCACACCTCAAACTGCAAAAG	31.7	X------T--CAnnnnnn------------
NC_000001.9	CTTTTGCAGTTTGAGGTGTGACAGCAAAA	31.7	------------nnnnnnTG--A------X
NC_000001.9	TTTTGCAGTTTGAAGTGCAACAACAAAA	31.7	X-----------nnnnnnC---AA-----X
NC_000001.9	TTTTGTTGTTGCACTTCAAACTGCAAAA	31.7	X-----TT---Gnnnnnn-----------X
NC_000001.9	TTTTGCAGTTTATTTTAAAACT	31.7	X-----------nnnnnn-----X??????
NC_000001.9	AGTTTTAAAATAAACTGCAAAA	31.7	??????X-----nnnnnn-----------X
NC_000001.9	TTTTGCAGTTTGAAATGCCACAGCAAAA	31.7	X-----------nnnnnnCC--A------X
NC_000001.9	TTTTGCTGTGGCATTTCAAACTGCAAAA	31.7	X------T--GGnnnnnn-----------X
NC_000001.9	TTTTGCAGTTTGAGGTGTGACAGCAAAA	31.7	X-----------nnnnnnTG--A------X
NC_000001.9	TTTTGCTGTCACACCTCAAACTGCAAAA	31.7	X------T--CAnnnnnn-----------X
NC_000001.9	TTTTGCTGTCACACCTCAAACTGCAAAA	31.7	X------T--CAnnnnnn-----------X
NC_000001.9	TTTTGCAGTTTGAGGTGTGACAGCAAAA	31.7	X-----------nnnnnnTG--A------X
NC_000018.8	TTTGCAGTTTGGTGATAAAATGCAAA	31.7	?X----------nnnnnn---A------X?
NC_000018.8	TTTGCATTTTATCACCAAACTGCAAA	31.7	?X------T---nnnnnn----------X?
NC_000018.8	TTTTGCTGTTGTACCTCACACTGCAAAAG	31.7	X------T---Gnnnnnn-C----------
NC_000018.8	CTTTTGCAGTTTGAGGAGCAATAGCAAAA	31.7	------------nnnnnnC--TA------X
NC_000018.8	TTTTGCTATTGCTCCTCAAACTGCAAAAG	31.7	X------TA--Gnnnnnn------------
NC_000018.8	CTTTTGCAGTTTGAGGTGCGACAGCAAAA	31.7	------------nnnnnnCG--A------X
NC_000018.8	TTTTGCTGTCGCACCTCAAACTGCAAAAG	31.7	X------T--CGnnnnnn------------
NC_000018.8	AGTTTTCTGAAAAACTGCAAAA	31.7	??????X-----nnnnnn-----------X
NC_000018.8	TTTTGCAGTTTTTCAGAAAACT	31.7	X-----------nnnnnn-----X??????
NC_000018.8	CTTTTGCAGTTTAATAATAAAC	31.7	------------nnnnnn----X???????
NC_000018.8	GTTTATTATTAAACTGCAAAAG	31.7	???????X----nnnnnn------------
NC_000018.8	TTTTGCAGTTTCTTAATAAACT	31.7	X-----------nnnnnn-----X??????
NC_000018.8	AGTTTATTAAGAAACTGCAAAA	31.7	??????X-----nnnnnn-----------X
NC_000018.8	TTTTGCTGTCACACCTCAAACTGCAAAA	31.7	X------T--CAnnnnnn-----------X
NC_000018.8	TTTATCTGTTTTTGCTGAAACTGCAAAA	31.7	X---AT-T----nnnnnn-----------X
NC_000018.8	TTTTGCAGTTTCAGCAAAAACAGATAAA	31.7	X-----------nnnnnn----A-AT---X
NC_000013.9	TTTTGCTGTTGTCCCTCAAACTGCAAAA	31.7	X------T---Gnnnnnn-----------X
NC_000013.9	TTTTGCAGTTTGAGGGACAACAGCAAAA	31.7	X-----------nnnnnnC---A------X
NC_000013.9	TTTTGCTGTCACACTTCAAACTGCAAAAG	31.7	X------T--CAnnnnnn------------
NC_000013.9	CTTTTGCAGTTTGAAGTGTGACAGCAAAA	31.7	------------nnnnnnTG--A------X
NC_000013.9	CTTTTGCAGTTTCCTGGAGAACTGAGAAA	31.7	------------nnnnnnG-----AG---X
NC_000013.9	TTTCTCAGTTCTCCAGGAAACTGCAAAAG	31.7	X---CT-----Cnnnnnn------------
NC_000013.9	CTTTTGCAGTTTGAGATGCAACAACAAAA	31.7	------------nnnnnnC---AA-----X
NC_000013.9	TTTTGTTGTTGCATCTCAAACTGCAAAAG	31.7	X-----TT---Gnnnnnn------------
NC_000013.9	CTTTTGCAGTTTCAGGTGTGACAGCAAAA	31.7	------------nnnnnnTG--A------X
NC_000013.9	TTTTGCTGTCACACCTGAAACTGCAAAAG	31.7	X------T--CAnnnnnn------------
NC_000013.9	TTTTTCTTTTTTTCTGAAAACTGCAAAAG	31.7	X----T-TT---nnnnnn------------
NC_000013.9	CTTTTGCAGTTTTCAGAAAAAAAGAAAAA	31.7	------------nnnnnn---AA-A----X
NC_000013.9	AGTTTTCAGACAAACTGCAAAA	31.7	??????X-----nnnnnn-----------X
NC_000013.9	TTTTGCAGTTTGTCTGAAAACT	31.7	X-----------nnnnnn-----X??????
NC_000013.9	AGTTTTATAAGAAACTGCAAAA	31.7	??????X-----nnnnnn-----------X
NC_000013.9	TTTTGCAGTTTCTTATAAAACT	31.7	X-----------nnnnnn-----X??????
NC_000013.9	AGTTTTGTAAGAAACTGCAAAA	31.7	??????X-----nnnnnn-----------X
NC_000013.9	TTTTGCAGTTTCTTACAAAACT	31.7	X-----------nnnnnn-----X??????
NC_000013.9	TTTTGCAGTTTGAGGTTCAACAGGAAAA	31.7	X-----------nnnnnnC---A-G----X
NC_000013.9	TTTTCCTGTTGAACCTCAAACTGCAAAA	31.7	X----C-T---Gnnnnnn-----------X
NC_000013.9	TTTTGCAGTTTGAGGTGTGACAGCAAAA	31.7	X-----------nnnnnnTG--A------X
NC_000013.9	TTTTGCTGTCACACCTCAAACTGCAAAA	31.7	X------T--CAnnnnnn-----------X
NC_000022.9	TTTGCAGTTTGAGGTACAACAGCAAAA	31.7	?X----------nnnnnnC---A------X
NC_000022.9	TTTTGCTGTTGTACCTCAAACTGCAAA	31.7	X------T---Gnnnnnn----------X?
NC_000022.9	TTTTGCAGTTTTGAATAAAGCTGC	31.7	X-----------nnnnnn--G----X????
NC_000022.9	GCAGCTTTATTCAAAACTGCAAAA	31.7	????X----C--nnnnnn-----------X
NC_000016.8	AGTTTTGCAGAAAACTGCAAAAG	31.7	??????X-----nnnnnn------------
NC_000016.8	CTTTTGCAGTTTTCTGCAAAACT	31.7	------------nnnnnn-----X??????
NC_000016.8	TTTACAATTTTAATGACAACTGCAAAAG	31.7	?X---A--A---nnnnnnC-----------
NC_000016.8	CTTTTGCAGTTGTCATTAAAATTGTAAA	31.7	-----------Gnnnnnn---T--T---X?
NC_000016.8	CTTTTGCAGTTTCTACAAAAAC	31.7	------------nnnnnn----X???????
NC_000016.8	GTTTTTGTAGAAACTGCAAAAG	31.7	???????X----nnnnnn------------
NC_000019.8	GTTTTTGTACAAACTGCAAAAG	31.7	???????X----nnnnnn------------
NC_000019.8	CTTTTGCAGTTTGTACAAAAAC	31.7	------------nnnnnn----X???????
